# Elevated Detection of Dual Antibody B Cells Identifies Lupus Patients With B Cell-Reactive VH4-34 Autoantibodies

**DOI:** 10.3389/fimmu.2022.795209

**Published:** 2022-02-04

**Authors:** Jacob N. Peterson, Susan A. Boackle, Sophina H. Taitano, Allison Sang, Julie Lang, Margot Kelly, Jeremy T. Rahkola, Anjelica M. Miranda, Ryan M. Sheridan, Joshua M. Thurman, V. Koneti Rao, Raul M. Torres, Roberta Pelanda

**Affiliations:** ^1^ Department of Immunology and Microbiology, University of Colorado School of Medicine, Aurora, CO, United States; ^2^ Division of Rheumatology, University of Colorado School of Medicine, Aurora, CO, United States; ^3^ Mucosa and Vaccine Research Program Colorado, Division of Infectious Diseases, University of Colorado School of Medicine, Aurora, CO, United States; ^4^ Rocky Mountain Regional Veteran Affairs Medical Center, Aurora, CO, United States; ^5^ RNA Bioscience Initiative, University of Colorado Anschutz Medical Campus, Aurora, CO, United States; ^6^ Division of Nephrology and Hypertension, University of Colorado School of Medicine, Aurora, CO, United States; ^7^ National Institute of Allergy and Infectious Diseases, National Institute of Health, Bethesda, MD, United States; ^8^ Department of Immunology and Genomic Medicine, National Jewish Health, Denver, CO, United States

**Keywords:** B cell, antibodies, SLE, lupus, autoimmunity, VH4-34, single cell RNA-seq

## Abstract

About 5% of B cells in healthy mice and humans are allelically or isotypically included and hence co-express two different antibodies. In mice, dual antibody B cells (B_2R_) expand with systemic autoimmunity, co-express autoreactive and non-autoreactive antibodies, and participate in immune responses, but this phenomenon is strain dependent. This study was developed with two goals: 1) to establish the contribution of TLR and IFN receptor signaling to the development of germinal center B cells that express two antibodies in MRL/*lpr* mice; and 2) to determine whether B_2R_ B cells are increased and particularly activated in a subset of adult patients diagnosed with systemic lupus erythematosus (SLE). Results from the MRL/*lpr* studies indicate that the enhanced differentiation of dual-κ B cells into germinal center B cells is due to a heightened response to TLR7 and TLR9 signaling, further fueled by an increased response to type II IFN. To understand the clinical and translational implications of our observations in mouse B_2R_ B cells, cohorts of SLE patients and healthy controls were recruited and evaluated for expression of dual BCRs. Results from flow cytometry and microscopy revealed supraphysiological frequencies of κ^+^λ^+^ B_2R_ cells in one fourth of the SLE patients. Abnormal numbers of κ^+^λ^+^ B cells correlated with higher frequencies of activated naïve B cells and age-associated B cells, and a lower proportion of “B cells that are naïve IgD^+^” (BND). However, results from single cell V(D)J sequencing demonstrated that these high κ^+^λ^+^ SLE patients harbored normal frequencies of κ^+^λ^+^ and other B_2R_ B cells. and we further show that their B cells were instead decorated by κ and λ VH4-34 autoantibodies. Thus, our findings indicate that elevated flow cytometric detection of isotypically-included B cells can identify patients with high titers of B cell-reactive VH4-34 autoantibodies and abnormal distribution of B cell subsets relevant to autoimmunity.

## Introduction

Allelic exclusion at the immunoglobulin (Ig) loci is a fundamental tenet of immunology that is considered essential for achieving immune responses that are specific and selective (1). Indeed, most circulating B cells carry allelically excluded Ig heavy (H) and light (L) chain loci, which ensures the expression of only one antibody of restricted specificity in each B cell. Defying the allelic exclusion tenet, some B cells (here named B_2R_) bear one or more allelically-included Ig loci and co-express two H or, most frequently, two L chains and, thus, two or more antibody specificities [reviewed in (2)]. Human B cells co-expressing two distinct Ig L chains produced by productively rearranged *Igκ* and *Igλ* loci (κ^+^λ^+^ B cells) have been previously described in peripheral blood of healthy individuals at a frequency of 0.2-0.5% of the total B cell population (3), a frequency which is similar to that reported in mice (4). Ig gene sequencing of human κ^+^λ^+^ B_2R_ cell clones revealed that a majority carry somatic mutations, indicating they have participated in immune responses (3).

To study B_2R_ cells in mice, we and others have benefited from a gene targeted mouse strain harboring one murine and one human *Igk* constant region allele and, thus, facilitating easy identification of B_2R_ cells by flow cytometry (5). Studies in these mice have determined that dual-κ B cells are 2-5% of all B cells, populate all B cell subsets, and participate in immune responses (6, 7). Interestingly, dual-κ B cells were found to be elevated in lupus-prone MRL and MRL/*lpr* mice whereby they accumulate with age and disease progression and are enriched in autoreactive clones specific for nuclear self-antigens (6, 8). In MRL(*lpr*) mice, moreover, dual-κ B cells are frequently activated relative to single-κ B cells, and they are particularly enriched in the germinal center (GC) and memory B cell subsets and in plasmablasts (8, 9). Furthermore, MRL/*lpr* dual-κ B cells display Toll-like receptor (TLR) and interferon (IFN) gene expression signatures that manifest with heightened *in vitro* cell expansion or activation in response to TLR7 and TLR9 ligands, and type I and II IFNs, respectively (9). However, it currently remains unresolved how each of these signals contribute to the development of dual-κ GC B cells. Here we show that TLR signaling together with IFNγ signaling drive the enhanced differentiation of MRL/*lpr* dual-κ B cells into GC B cells.

Interestingly, not every lupus-prone mouse strain harbors an increased frequency of B_2R_ cells. In fact, a study of NZB/NZW F1 mice, which have penetrance and kinetics of lupus disease similar to MRL but a different genetic background, found no differences in B_2R_ cell frequency relative to healthy mice (10). This suggests that specific autoimmune-prone genetic variants, most likely acting at the stage of central B cell tolerance, must be present to support the increased generation and/or selection of B_2R_ cells. Based on these findings we posited that B_2R_ cells may be increased in a subset of human Systemic Lupus Erythematosus (SLE) patients. Humans have similar frequencies of κ^+^ and λ^+^ B cells and, thus, similar opportunity for generating B cells with either two κ, two λ, or one κ and one λ chains. Because it is not possible to differentiate by antibody staining B cells co-expressing two λ or two κ chains, in this study we investigated κ^+^λ^+^ B_2R_ cells in adults diagnosed with SLE in comparison to healthy controls. In our patient cohort, about one fourth of SLE subjects exhibited a higher frequency of B_2R_ B cells by flow cytometry and microscopy, a result in line with a previous UK study (11). However, single cell V(D)J sequencing was unable to support this finding. Instead, results from our studies show that the elevation in κ^+^λ^+^ B cells resulted from the decoration of VH4-34 (κ and λ) autoantibodies, which are known to bind carbohydrate molecules on the surface of B cells, and to be elevated in in a large fraction of SLE patients (12–14). In conclusion, higher numbers of κ^+^λ^+^ B cells identify a subset of SLE patients with high sera VH4-34 autoantibodies and other B cell-related abnormalities.

## Material and Methods

### Human Subjects and Sample Preparation

Human studies were approved by the University of Colorado Institutional Review Board and were performed in accordance with the Declaration of Helsinki. Peripheral blood samples from 18 adult SLE patients were obtained after the recruitment and consent of subjects at the University of Colorado Health (UCH) Rheumatology clinics Clinical data was collected and SLEDAI scores were calculated on either the day of the blood draw or on the date of the clinical visit closest to the blood draw. Blood samples from 18 healthy controls were from two sources: 1) de-identified discarded samples from the Colorado Bonfils (now Vitalant) Blood bank isolated *via* plateletpheresis leukoreduction filters; 2) healthy adult volunteers recruited and consented at the University of Colorado Anschutz Medical Campus. Frozen peripheral blood mononuclear cells (PBMCs) from 10 Autoimmune Lymphoproliferative Syndrome (ALPS) patients (age 3-24 years) were obtained from NIAID (Dr. V. Koneti Rao) as de-identified samples. Peripheral Blood Mononuclear Cells (PBMCs) were separated over a Ficoll-Plaque density gradient and analyzed by flow cytometry either fresh or after cryopreservation. To cryopreserve, PBMCs were resuspended in 10% DMSO, 90% FBS, and stored at –80°C for short term or in liquid nitrogen for long-term storage. Sera was recovered from each sample and stored at –20°C until analysis. Genomic DNA was extracted from each PBMC sample for diagnostic PCR of the PTPN22 alleles encoding the R620 or W620 variants (15).

### Mice

MRL/*lpr-Igk^m/h^* mice have been previously described (8, 9). Male and female mice 6-16 weeks of age were used in experiments. For some analyses, groups of 6-7 week old MRL/*lpr-Igk^m/h^* mice were first injected i.p. with 200 μg of CpG ODN 1826 (IDT) twice a week for four weeks, or with 0.9 μg or 9.0 μg of IFNα (BioLegend) every other day for one week. Control mice were injected with equal volume of PBS. Mice were housed in Specific Pathogen Free conditions at the University of Colorado Anschutz Medical Campus (UCD-AMC). All animal procedures were approved by the UCD-AMC Institutional Animal Care and Use Committee and carried out in accordance with approved guidelines.

### Culture, Sorting, and Flow Cytometric Analysis of Mouse B Cells

Untouched splenic B cells from MRL/*lpr-Igk^m/h^* mice were enriched by negative selection using anti-CD43 magnetic beads and an AutoMACS Pro Separator (both from Miltenyi Biotech). Enriched B cells were cultured immediately or after additional fluorescence activated cell sorting. For sorting, enriched B cells were stained with PNA (Vector Laboratories), antibodies to CD3 (145-2C11), CD43 (eBioR2/60), and GL7 (GL7), and Fab’ antibody fragments to human Igκ (goat polyclonal, Protos Immunoresearch) and mouse Igκ (187.1, generated in house). Single-κ (hIgκ^+^ or mIgκ^+^) and dual-κ (hIgκ^+^mIgκ^+^) non-germinal center B cells were sorted after excluding CD3^+^, CD43^+^, GL7^+^, and PNA^+^ cells. The B cells were sorted directly into complete RPMI medium with 3% heat-inactivated FBS and 10 ng/ml BAFF (R&D System). Enriched or sorted B cells were cultured for 60 hours in complete RPMI, 3% FBS, 0.05 mM β-ME, 10 ng/ml BAFF with the addition or not of 1 μg/ml R848 (Invivogen), 1000 U/ml IFNα (BioLegend), and 50 ng/ml IFNγ (R&D System). For cell staining and flow cytometric analyses of *ex-vivo* spleen cells and cultured B cells, cells were first incubated with anti-FcγRII/III 2.4G2 antibodies (homemade) and then stained with PNA and antibodies to surface CD19, CD38, GL7, human Igκ, and mouse Igκ. For BCL6 detection, after surface staining, cells were fixed and permeabilized using the True-Nuclear kit (BioLegend) and then stained with antibodies to BCL6 (K112-91, BioLegend). Dead cells were excluded by staining with Ghost dyes (Cell Signaling). Stained cells were analyzed on a BD LSRFortessa (BD Biosciences), and with FlowJo v10.7.1 software (FlowJo, LLC/BD, Ashland, OR, USA). In all analyses, cells were first serially gated as single cells, live cells, and lymphocytes.

### Flow Cytometric Analysis of Human Cells

Human PBMCs were processed for flow cytometric analyses fresh or after thawing, and in some assays after an additional 30 minutes incubation at 4°C in undiluted human sera. Frozen PBMCs samples were quickly thawed at 37°C and centrifuged over a Ficoll density gradient at room temperature to remove dead cells before further processing. PBMCs were stained in staining buffer PBS, 1% BSA, 0.1% NaN_3_ with antibodies (clone number in parenthesis) specific for the following antigens: CD3 (HIT3a), CD11c (3.9), CD14 (63D3), CD19 (SJ25C1), CD20 (2H7), CD21 (Bu32), CD24 (ML5), CD27 (O323 or L128), CD33 (P67.6), CD38 (HB7), CD86 (IT2.2), IgD (IA6-2), Igκ (MHK-49), Igλ (MHL-38), IgM (MHM-88), IgG (G18-145), Tbet (4B10), purchased from either BioLegend or BD. CD3, CD14, and CD33 were stained in the same channel and used to gate out (“Dump”) non-B cells and nonspecific binding of antibodies. Isotype control antibodies for Igκ and Igλ staining were clones MOPC-21 (mouse IgG1,κ) and MOPC-173 (mouse IgG2a,κ), respectively. Unconjugated anti-VH4-34 9G4 antibodies (IgM Biosciences) were revealed with fluorescent goat anti-rat IgG (Southern Biotech). Dead cells were revealed with either Zombie Green (Biolegend) or SYTOX Red (ThermoFisher). Biolegend True-Nuclear fix buffer and perm buffer were used for the simultaneous staining of nuclear Tbet and cytoplasmic Igκ and Igλ. Stained cells were analyzed on a BD LSRFortessa (BD Biosciences), besides ALPS samples, which were analyzed on a CyAn flow cytometer (Beckman Coulter). Data were analyzed with FlowJo v10.7.1 software (FlowJo, LLC/BD, Ashland, OR, USA). In all B cell analyses, cells were first serially gated as single cells, live cells, and lymphocytes.

### Fluorescent Microscopy and ImageStream Analyses

Microscopic analysis of CD19, Igκ, and Igλ expression in human B cells was performed by fluorescent microscopy and ImageStream. Human PBMCs were stained with anti-CD19-APC, anti-Igλ-PE, and anti-Igκ-FITC (for cytospin) or anti-Igκ-BV421 (for ImageStream). Stained cells were washed in staining buffer PBS, 1% BSA, 0.1% NaN_3_ and further processed for analysis. For microscopy, 1x10^6^ stained cells, resuspended in 150 μl of staining buffer, were loaded in a cytospin centrifuge and spun at 1,500 rpm for 5 minutes into each microscope slide. After drying the slides in air for 10 minutes, one drop of cytoseal mounting media was added and a cover slip applied. Cytospin slides were analyzed, and images collected using an Eclipse TE 2000 microscope and NIS Elements version 4.2 software (Nikon). Stained cells were also analyzed by ImageStream (Amnis, Seattle, WA) at a concentration of 1x10^6^ cells/ml and under blinded conditions. ImageStream data were analyzed using IDEAS 6.2 Software (Amnis).

### ELISA

Sera VH4-34 IgM and IgG titers were measured by ELISA. Briefly, 96-well Nunc-Immuno MaxiSorp plates (Thermo Fisher Scientific) were coated overnight at 4°C with 1 µg/ml of rat anti-human VH4-34 9G4 antibodies (IgM Biosciences). The following day, plates were washed four times in PBS, 0.5% Tween-20 and blocked for 2h at room temperature with PBS 1% BSA, 0.1% NaN_3_. Plates were then washed three times and plasma samples added to the plates at twofold serial dilutions starting at 1:250 (IgM) or 1:100 (IgG). Plates were then incubated for 2h at 37°C or overnight at 4°C and subsequently washed four times. Human VH4-34 IgM and IgG on plates were then detected by incubating for 2h at 37°C with alkaline phosphatase (AP)-conjugated mouse anti-human IgM (Southern Biotechnologies Associates, #2020-04) or AP-conjugated mouse anti-human IgG (Southern Biotechnologies Associates, #2014-04) diluted at 1:1000. After washing four times, plates were developed by the addition of AP substrate (Sigma-Aldrich) solubilized in 0.1 M diethanolamine and 0.02% NaN_3_. Light absorbance was measured at OD_405_ multiple times with a VersaMax plate reader (Molecular Devices). Data were analyzed with the SoftMax Pro software v6.4.2. Titers were calculated relative to an internal human Ig standard.

### Single Cell V(D)J Sequence Analyses

Frozen human PBMCs were rapidly thawed in a 37°C water-bath and dead cells were removed with the Dead Cell Removal kit and an AutoMACS Pro Separator (both from Miltenyi Biotech). Untouched B cells were then isolated with the B cell Isolation kit II (Miltenyi Biotech) and resuspended in PBS, 0.04% BSA at 7-11x10^5^ cells/ml. The cells were immediately processed by the CU-AMC Genomics & Microarray Core facility on a 10X Chromium Controller (10X Genomics) with a targeted recovery of 5,000 cells per sample. The Chromium Next GEM Single Cell 5’ Library and V(D)J Enrichment Kit for Human B Cells (version 1.1) was used to construct V(D)J-enriched libraries (following manufacturer’s instructions). V(D)J libraries were sequenced on a NovaSEQ 6000 (Illumina) with 2x150 base pair read lengths. FASTQ files from the V(D)J libraries were processed and aligned using the cellranger vdj pipeline (v3.1.0, 10X Genomics) and the reference file refdata-cellranger-vdj-GRCh38-alts-ensembl-3.1.0. Immunoglobulin heavy and light chain gene sequences of single B cells were analyzed using the Loupe VDJ Browser (v4.1.0, 10X Genomics) to generate VH usage reports for each sample. The heavy and light chain paired clonotype sequences were first analyzed by Numbers (v11.1) to create Excel (v16.53) spreadsheets containing a list of clonotypes bearing only one heavy chain and that were either IgM/D or Ig class switched. Excel was further used to sort each sample clonotypes in relation to their expression of two light chains or of VH4-34. The clonotype analyses depicted in Figures and Tables were performed in Excel and GraphPad Prism (v9.1.2). The data discussed in this publication have been deposited in NCBI’s Gene Expression Omnibus (16) and are accessible through GEO Series accession number GSE183051 (https://www.ncbi.nlm.nih.gov/geo/query/acc.cgi?acc=GSE183051).

### Statistics

Statistical significance evaluation was performed with GraphPad Prism software (v9.1.2). Student t-test was used for groups with normal distribution. The non-parametric Mann-Whitney test was used for when groups did not pass a normality test. Simple linear regression analysis was used for scatter plot correlations. The level of significance was: *p<0.05; **p<0.01; ***p<0.001; ****p<0.0001. Data in graphs is represented as mean ± SD.

## Results

### Germinal Center B Cell Differentiation of Mouse Single and Dual-κ B Cells Following TLR and IFN Receptor Stimulation

We have previously shown that in lupus-prone MRL/*lpr* mice, dual-κ B cells mount enhanced antibody responses to a T cell-dependent immunization when compared to single-κ B cells, a difference amplified in the presence of the TLR9 agonist CpG (9). Relative to single-κ cells, dual-κ B cells showed superior expansion but not activation to both CpG and the TLR7 agonist R848, *in vitro*. In contrast, treatment with type I or II IFNs led to increased activation, but not expansion, of dual-κ B_2R_ cells (9). To better understand how these innate signals contribute to the differential response of MRL/*lpr* B_2R_ cells, we compared *in vivo* and *in vitro* the ability of single-κ and dual-κ B cells to differentiate into germinal center (GC) B cells.

**Figure 1:**
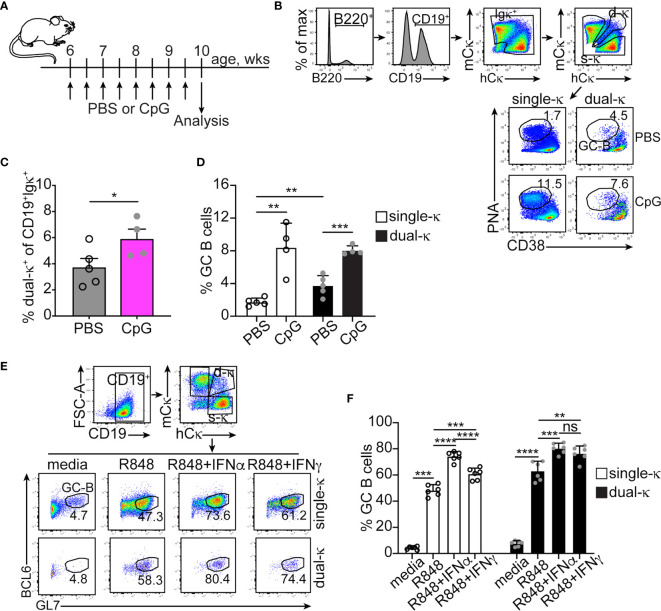
IFNα and IFNγ synergize with TLR7 and TLR9 to promote the differentiation of single-κ and dual-κ MRL/*lpr* B cells into GC B cells. **(A)** Schematic of the *in vivo* treatment of 6 week old MRL/*lpr* mice with the TLR9 ligand CpG. **(B)** Flow cytometric analysis and gating strategy of splenic B cells from MRL/*lpr* mice injected with either PBS or CpG. The cells were serially gated to measure the frequency of PNA^high^CD38^low^ GC B cells within the single-κ and dual-κ B cell populations. **(C, D)** Frequencies of dual-κ B cells and PNA^high^CD38^low^ GC B cells within the single-κ and dual-κ cell populations, in MRL/*lpr* mice treated with either PBS or CpG. N=4, analyzed in two independent experiments. Data is shown as mean+SD. Statistical analysis was performed with a one-tailed unpaired t-test. **(E)** Flow cytometric analysis of B cells enriched from the spleen of MRL/*lpr* mice (8-15 weeks of age) and cultured for 60 hours in media alone or with R848, R848+IFNα, or R848+IFNγ. The cells were serially gated to measure BCL6^+^GL7^high^ GC B cells within the single-κ and dual-κ B cell populations. **(F)** Frequencies of BCL6^+^GL7^high^ GC B cells within the single-κ and dual-κ B cell populations of MRL/*lpr* B cells cultured as described in **(E)**. N=6, analyzed over one experiment (data from a similar experiment are in **Supplementary Figures S1** **D–F**). Data are shown as mean+SD. Statistical analysis was performed with a one-tailed unpaired t-test. *p ≤ 0.05; **p < 0.01; ***p < 0.001; ****p < 0.0001; ns, not significant.

*In vivo* treatment of young MRL/*lpr* mice with CpG led to a small expansion of dual-κ B cells over single-κ B cells (**Figures 1A–C**), in line with previous *in vitro* findings (9). We also observed a significant increase in the percent of GC B cells in both the single-κ and dual-κ cell subsets (**Figures 1B, D**). In contrast, treatment of MRL/*lpr* mice with IFNα did not increase the frequency of GC B cells (**Supplemental Figures S1A–C**). To further explore the contribution of these innate signals to the differentiation of GC B cells, enriched B cells (**Figures 1E, F**) or sorted single and dual-κ PNA^–^GL7^–^ (non-GC) B cells (**Supplementary Figures S1D–F**) were cultured for 2-3 days in the presence or absence of R848 and type I and II IFNs. In these cultures, TLR7 stimulation led to a dramatic increase in the proportion of GC B cells in both the single-κ and dual-κ B cell populations (**Figure 1F** and **Supplementary Figure S1F**). The proportion of GC cells was further enhanced by the addition of either type I or type II IFNs. However, the synergistic response of dual-κ B cells to R848 and IFNγ was significantly higher than that of single-κ B cells.

These data suggest that in MRL/*lpr* mice, the enhanced ability of dual-κ B cells over single-κ B cells to respond to (self or foreign) antigens is primarily due to a heightened sensitivity to TLR7 and/or TLR9 ligands, further fueled by an increased response to type II IFN.

### A Colorado Cohort of SLE Patients Displays Elevated Frequencies of Ig κ/λ B_2R_ Cells

**Figure 2:**
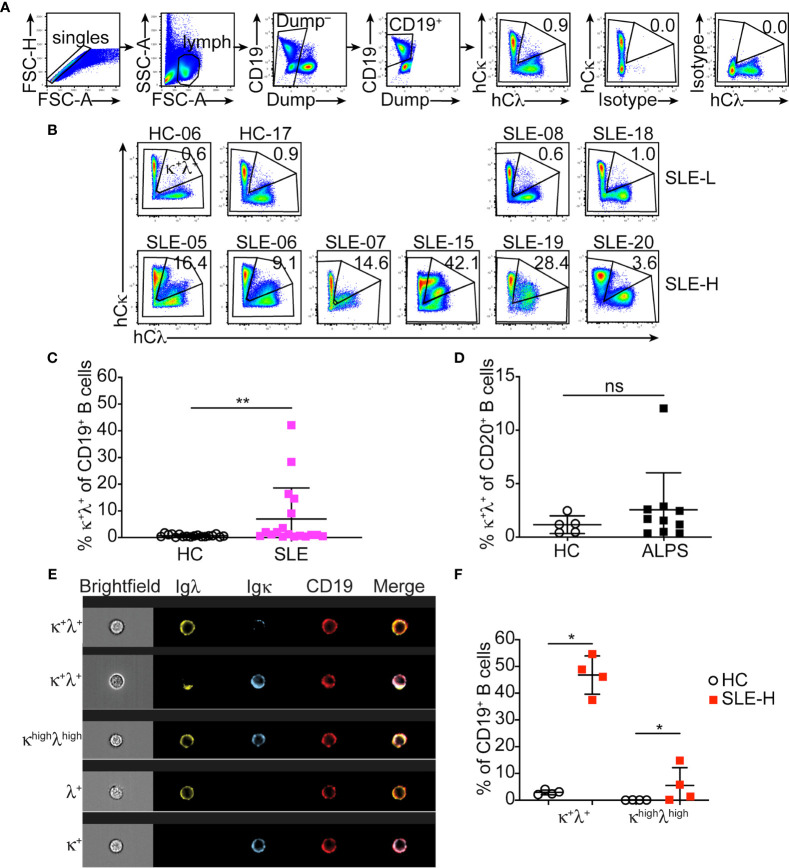
Detection of κ^+^λ^+^ B cells in SLE patients and healthy controls. **(A)** Representative flow cytometric analysis and gating strategy of CD19^+^ B cells from the blood of healthy controls and SLE patients. The top two plots to the right demonstrate isotype control staining for Igκ and Igλ. **(B)** Igκ and Igλ expression on CD19^+^ B cells from representative healthy controls (HC) and SLE-L patients (top), and from all SLE-H patients with high κ^+^λ^+^ cells (bottom). Data were collected over a total of 18 independent analyses. **(C)** Frequency of κ^+^λ^+^ cells within the blood CD19^+^ B cell population in each healthy control and SLE patient. N=18 in each group. **(D)** Frequency of κ^+^λ^+^ cells within the blood CD20^+^ B cell population in healthy controls (N=5) and ALPS patients (N=10). Data were collected over 3 independent analyses. **(E)** Representative Image-Stream analysis of PBMCs stained for CD19 (red), Igλ (green), and Igκ (blue). The top three rows show example of CD19^+^ B cells that express both κ and λ; the cell shown in the third row was defined as bright for both κ and λ (κ^high^λ^high^). Bottom two rows are examples of single λ^+^ or κ^+^ cells. Brightfield, individual fluorescence, and merge fluorescence are shown for each cell. **(F)** Frequency of all κ^+^λ^+^ cells and of bright (κ^high^λ^high^) cells analyzed by Image-Stream among CD19^+^ cells from the blood of N=4 healthy controls and N=4 SLE patients with high κ^+^λ^+^ cell frequency. The analysis was performed on an average of 2263 ± 740 CD19^+^ cells (range 1470-3074) per sample. Data are from one experiment. In all graphs, each symbol is an individual, lines represent mean ± SD, and statistical analysis was performed with a Mann-Whitney *U* test. *p < 0.05; **p < 0.01; ns, not significant.

The autoimmune-mediated expansion of B_2R_ B cells we reported in MRL and MRL/*lpr* mice is not present in NZB/NZW mice (10), a different model of lupus-like disease. This has led us to postulate that a subset of SLE patients may display increased frequencies of B_2R_ B cells. Human B cells harbor an approximate 1:1 ratio of κ and λ cells. Thus, we developed a flow cytometric protocol to measure B cells co-expressing κ and λ light chains (**Figure 2A**).

SLE patients (N=18) were recruited into our study at the Rheumatology Clinic of the University of Colorado Health hospital. Healthy controls (N=18), with similar distribution of sex, age, race and ethnicity (**Supplementary Figure S2A**), were either recruited at the University of Colorado Anschutz Medical Campus or were de-identified peripheral blood samples obtained from a Colorado blood bank. Peripheral blood B cells from lupus patients and healthy controls were analyzed to measure frequency of κ^+^λ^+^ B_2R_ cells in the total B cell population (**Figure 2A**). The frequency of κ^+^λ^+^ B cells within the circulating B cell population of healthy individuals was consistently less than 2% (mean ± SD=0.73 ± 0.47; **Figures 2B, C**). Most individuals diagnosed with SLE (referred to as SLE-L hereafter) displayed frequencies of B_2R_ cells comparable to healthy controls. However, about one fourth of SLE patients (referred to as SLE-H) showed significantly higher frequencies of κ^+^λ^+^ B cells (defined as greater than 3 times the SD over the mean of healthy controls), with frequencies ranging from about 4% to 42% of all B cells (**Figures 2B, C**). These findings are consistent with a study of a UK SLE cohort (11). Upon unblinding individual Systemic Lupus Erythematosus Disease Activity Index (SLEDAI) scores, SLE-H patients were found to be distributed in higher proportion among the patients with SLEDAI scores ≥6, than in the group with SLEDAI ≤4, but this difference was not significant (**Supplementary Figure S2B**). SLE-L and SLE-H patients did not display significant difference in age distribution at diagnosis or time of analysis (**Supplementary Figure S2C**). There was also no remarkable difference in other characteristics of disease (e.g., kidney disease, presence of autoantibodies) and in treatments (**Supplementary Table S1**), although the group size was insufficiently powered for this analysis. Furthermore, all SLE patients carried the normal PTPN22 genetic variant (R620), and not the R620W variant associated with a more severe development of autoimmunity (**Supplementary Table S1**).

Our mouse studies have previously shown that in MRL mice the proportion of dual-κ B cells is amplified by the presence of the *lpr* Fas mutation (8). To test this in humans, we analyzed B cells from a cohort of patients with Autoimmune Proliferative Syndrome (ALPS (17);) carrying *FAS* inactivating mutations (**Supplementary Table S2**). Our analyses demonstrate that inactivation of *FAS* does not generally lead to higher frequencies of κ^+^λ^+^ B cells in humans as only one of the ten ALPS samples showed abnormal frequency of κ^+^λ^+^ B cells (**Figure 2D**).

To confirm that the Igκ and Igλ dual expression we observed by flow cytometry in SLE-H patients was genuine, we analyzed PBMC samples from a subset of SLE-H and healthy controls by microscopy and, specifically, by cytospin (**Supplementary Figure S2D**) and Image Stream (**Figures 2E, F**). Results from both analyses confirmed a higher proportion of κ^+^λ^+^ B cells in the selected SLE patients.

In summary, about one fourth of a cohort of adult SLE patients at the University of Colorado Health display a greater frequency of B cells harboring both κ and λ Ig light chains.

### Distribution of κ^+^λ^+^ Cells Among Human B Cell Subsets

**Figure 3:**
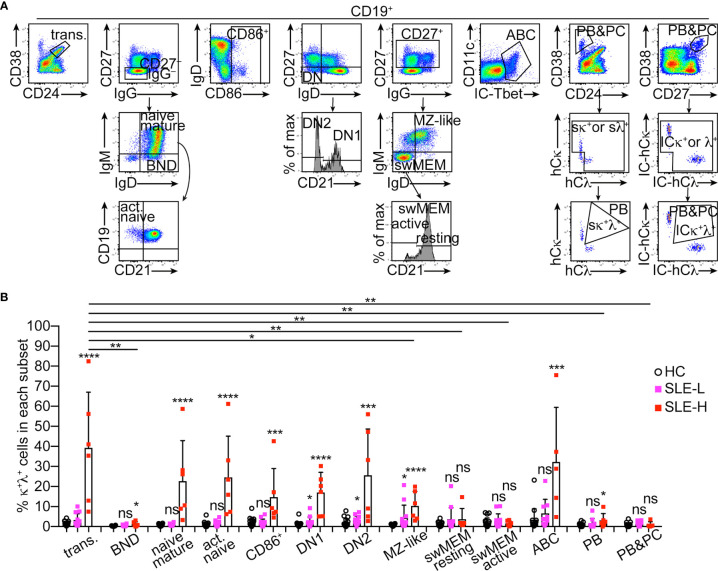
Distribution of κ^+^λ^+^ B cells among human B cell subsets. **(A)** Representative flow cytometric analysis and gating strategy of human CD19^+^ B cell subsets in PBMCs. All B cell subsets, except for the CD38^high^CD27^high^ (PBs&PCs) subset representing plasmablasts (PBs) and plasma cells (PCs), were analyzed for surface (s) Igκ and Igλ expression, and the frequency of κ^+^λ^+^ cells was measured only on cells positive for sIgκ and/or sIgλ, as shown for the CD38vsCD24 plot. For the CD38^high^CD27^high^ (PBs&PCs) subset, Igκ and Igλ were measured after cell fixation and permeabilization and, therefore, include intracellular (IC) expression. **(B)** Frequency of κ^+^λ^+^ cells within each B cell subset from healthy controls (N=18), and from SLE patients with low (SLE-L, N=12) and high (SLE-H, N=6) frequency of κ^+^λ^+^ cells. Data were collected over a total of 18 independent analyses. Each symbol is a subject, and bars represent mean+SD. Differences between groups were analyzed by a two-tailed Mann-Whitney *U* test. Differences between the HC group and each of the SLE groups are reported on top of each SLE bar. Differences between the transitional B cell subset and the other B cell subsets only in the SLE-H group, are depicted with horizontal lines on top of graph, and only if significant. *p ≤ 0.05; **p < 0.01; ***p < 0.001; ****p < 0.0001; ns, not significant.

We next asked whether κ^+^λ^+^ B cells were equally distributed among major human B cell subsets. Antibody panels were used to identify the following CD19^+^ B cell subsets (as described in (18)) that expressed Ig κ and λ strictly on the cell surface (**Figure 3A**): transitional B cells (CD38^high^CD24^high^), ‘B cells that are naïve IgD^+^’ (BND) anergic cells (CD27^–^IgG^–^IgD^+^IgM^low^), mature resting naïve B cells (CD27^–^IgG^–^IgD^+^IgM^+^), activated naïve B cells (CD27^–^IgG^–^IgD^+^IgM^+^CD21^–^), total activated B cells (CD86^+^), double negative DN1 B cells (CD27^–^IgD^–^CD21^+^), DN2 B cells (CD27^–^IgD^–^CD21^–^), MZ-like B cells (CD27^+^IgM^+^IgD^+^), switched memory resting B cells (CD27^+^IgM^–^IgD^–^CD21^+^), switched memory activated/exhausted B cells (CD27^+^IgM^–^IgD^–^CD21^–^), age-associated B cells (ABC; Tbet^+^CD11c^+^), and plasmablasts (CD38^high^CD24^–^). In a separate panel, staining of surface markers was followed by intracellular (IC) staining of Igκ and Igλ to analyze a cell population including both plasmablasts and plasma cells (CD38^high^CD27^high^, IC-Igκ^+^/λ^+^).

In the SLE-H patients, κ^+^λ^+^ cells were observed at supraphysiological frequencies among transitional B cells, resting and activated mature naïve B cells, total activated B cells, DN1/2 B cells, MZ-like B cells, and ABCs (**Figure 3B**). When compared to the transitional B cell subset as a point of origin, the proportion of κ^+^λ^+^ cells did not significantly change through development in all these subsets besides in the MZ-like subset where it was reduced (**Figure 3B**). Interestingly, κ^+^λ^+^ B cells were significantly less abundant and, in fact, seen at frequencies similar or only slightly elevated relative to control, in all other B cell subsets, including BNDs, switched memory B cells, plasmablasts and plasma cells (**Figure 3B**). The MZ-like B cell fraction was unique in that the frequency of κ^+^λ^+^ B cells was significantly elevated in both SLE patient groups (**Figure 3B**). Thus, B_2R_ cells are unevenly distributed in B cell subsets of SLE patients.

### Differences in B Cell Subsets Among SLE Patients and Healthy Controls

**Figure 4:**
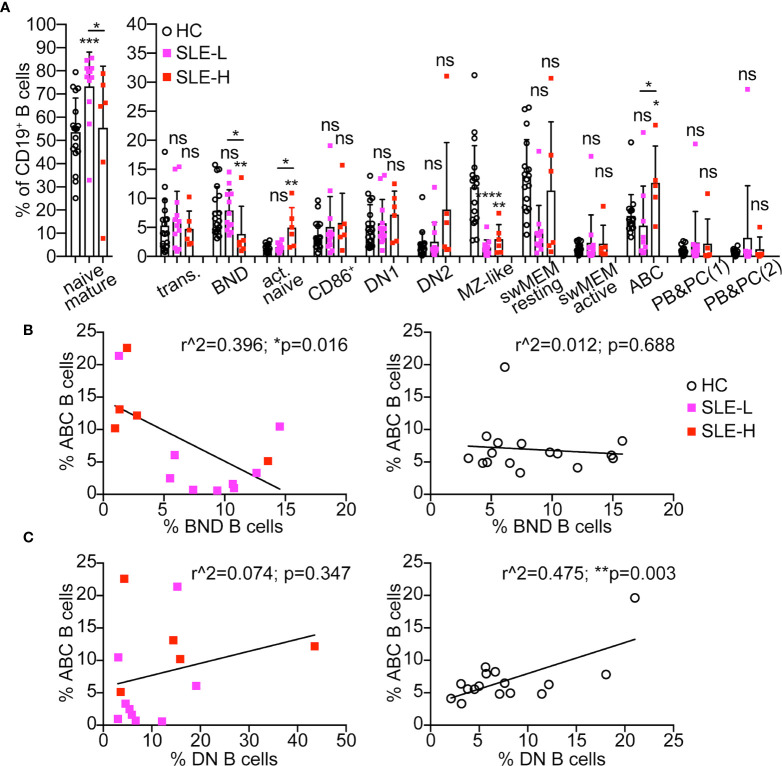
Distribution of B cell subsets in SLE patients and healthy controls. **(A)** Distribution of CD19^+^ B cells among B cell subsets in blood of healthy controls (HC, N=18) and SLE patients with low κ^+^λ^+^ (SLE-L, N=12) or high κ^+^λ^+^ (SLE-H, N=6) cells. Mature naïve B cells are graphed separately to visualize better group differences within the smaller B cell subsets. The B cell subsets were gated as shown in **Figure 3A**. PBs&PCs(1) were gated as CD38^high^CD24^low^, while PBs&PCs(2) were gated as CD38^high^CD27^high^. Data were collected over a total of 18 independent analyses. Each symbol is a subject, and bars represent mean+SD. Statistical analysis was performed with a Mann-Whitney *U* test. Statistical significance for differences between each SLE group and the healthy control group are shown on top of each SLE group bar. Differences between the SLE-L and SLE-H groups are shown with horizontal lines on top of the SLE bars and only if significant. **(B, C)** Scatter plot analyses of the percentage of ABCs (y-axis) relative to the percentage (on x-axis) of either BND **(B)** or DN **(C)** cells within the CD19^+^ B cell population of SLE patients (left graphs) and healthy controls (right graphs). Cells were analyzed as shown in **Figure 3A** and data were collected over the course of 18 independent analyses. Data were analyzed by Simple linear regression. *p ≤ 0.05; **p < 0.01; ***p < 0.0001; ns, not significant.

The subset distribution of B cells from SLE patients is known to be skewed relative to healthy controls, with a lower frequency of unswitched memory B cells (also known as MZ-like B cells), and a higher proportion of CD27^–^IgD^–^ DN B cells (reviewed in (19)). The population of DN B cells, which are precursors of antibody-secreting cells enriched in autoreactive clones, is elevated in 20-30% of all SLE patients, and in clear association with active disease and higher SLEDAI score, African-American descent, and adverse clinical outcomes (20–22). Upon measuring these B cell subsets in our human samples, we found that MZ-like B cells were indeed reduced in almost all SLE patients relative to controls, and both in SLE-L and SLE-H patient cohorts (**Figure 4A**). There was no significant difference in the frequencies of DN1 (CD21^+^), DN2 (CD21^–^), and of total DN B cells between healthy controls and SLE patients (**Figure 4A** and **Supplementary Figure S3A**). Nonetheless, we noticed that two SLE-L (17%) and three SLE-H (50%) patients displayed frequencies of DN2 B cells above the standard deviation of the healthy control group and, thus, at a proportion in line with previous reports for patients with inactive, mild and active (but not severe) disease (20–22).

BND, activated naïve (aNAV), and ABC (which are part of the DN2 population) are three additional B cell subsets enriched in autoreactive clones and, thus, of interest to autoimmunity. However, while BNDs are considered anergic (23, 24), aNAV and ABC cells are functional precursors of antibody-secreting cells (21, 25). Indeed, BNDs are decreased in pre and new onset T1 diabetes (26) and in early onset of autoimmune thyroid disease (27), while aNAV and ABCs are elevated with autoimmunity, including lupus (18, 21, 25). In accord with these previous reports, the frequencies of aNAV and/or ABC cells were increased in six SLE patients, and statistically in the SLE-H group (**Figure 4A**). Furthermore, ABCs negatively correlated with BNDs in SLE patients but not in healthy controls (**Figure 4B**). In contrast, ABCs positively correlated with DNs in healthy controls but not in SLE patients (**Figure 4C**). In addition, SLE-H (but not SLE-L) patients displayed a reduced frequency of BND cells (**Figure 4A**), and a significant negative correlation was observed between frequency of κ^+^λ^+^ B cells and proportion of BNDs in all SLE patients (**Supplementary Figure S3B**). However, no significant correlation was seen between the frequency of κ^+^λ^+^ B cells and those of ABCs and DNs (**Supplementary Figures S3C, D**).

In summary, SLE patients harboring a high frequency of κ^+^λ^+^ cells are generally characterized by a higher frequency of aNAVs and ABCs and a lower frequency of BNDs.

### Single Cell Analysis of Immunoglobulin Repertoire

Given the skewed distribution of κ^+^λ^+^ cells in B cell populations relevant to autoimmunity, we next examined the Ig gene repertoire in a subset of SLE-H patients relative to healthy controls. B cells from the blood of three SLE-H patients (SLE-07, SLE-015, SLE-019) and three healthy controls were processed for single cell V(D)J sequence analyses by the 10X Genomics platform. These analyses were performed on a newly acquired blood sample from the patients, a sample that still displayed supraphysiological numbers of κ^+^λ^+^ B cells (**Supplementary Figure S3E**). Live B cells were enriched from the blood before single cell analysis; B cell enrichment was less efficient for the SLE samples (**Supplementary Table S3**). Nevertheless, the number of recovered VDJ cell barcodes was proportional to the frequency of live B cells in all samples (**Supplementary Table S3**).

**Figure 5:**
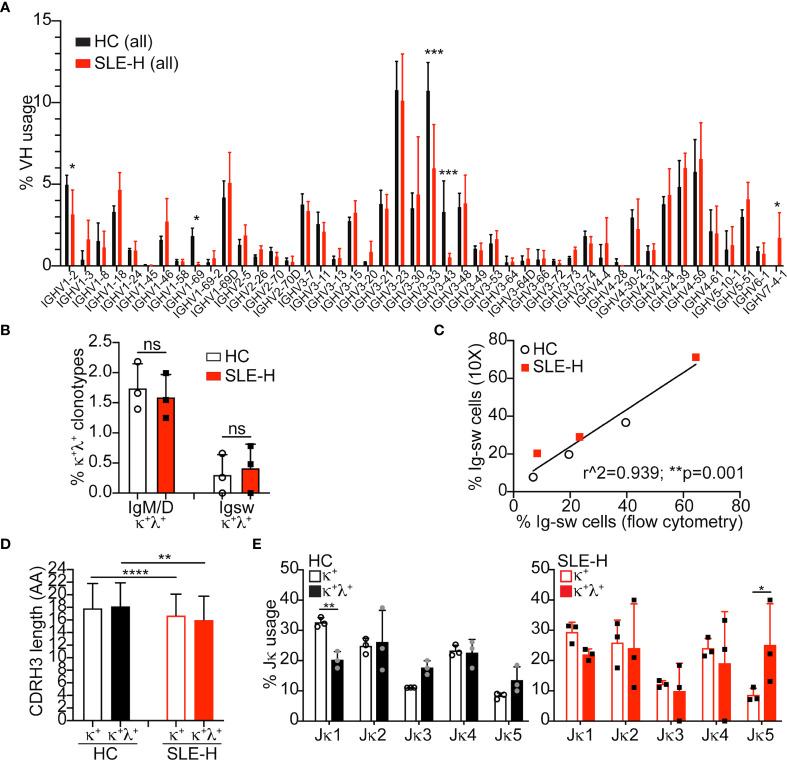
Single cell V(D)J-seq analysis of human B cells. **(A)** Average (mean+SD) Ig VH usage among all (switched and unswitched) B cell clonotypes from three healthy controls (HC) and three SLE-H patients. VH usage was obtained *via* 10X Genomics V(D)J-seq se analysis over two independent runs. The number of clonotypes per sample was between 887 and 5,418. Statistical analysis was performed with two-tailed unpaired t-tests. **(B)** Percentage of unswitched (IgM/D) or switched (Igsw) B cell clonotypes bearing one heavy chain and a κ and a λ light chains. Symbols represent individual subjects and bars are mean+SD. Statistical analysis was performed with two-tailed Mann-Whitney *U* tests. **(C)** Scatter plot analysis of the percentage of Ig switched (IgM^–^IgD^–^) B cells measured by 10X (y-axis) or flow cytometry (x-axis) in the same SLE-H and healthy control subjects. Data were analyzed by Simple linear regression. **(D, E)** Amino acid (AA) length of the heavy chain CDR3 sequence **(D)** and Jκ usage **(E)** in IgM/D (unswitched) κ^+^ and κ^+^λ^+^ clonotypes from healthy controls and SLE-H patients. Statistical analysis was performed with two-tailed unpaired t-tests. *p ≤ 0.05; **p < 0.01; ***p < 0.001; ****p < 0.0001; n.s., not significant (differences were not significant when lacking asterisks).

V(D)J sequences were analyzed among B cell clonotypes (i.e., cells with unique H and L chain pairs) displaying only one VH genes and at least one L chain gene. Analysis of VH usage among B cell clonotypes demonstrated a much lower usage of VH1-69, VH3-33, and VH3-43 genes, and a higher usage of VH7-4-1 in B cells from SLE patients compared to control B cells (**Figure 5A**). The B cell clonotypes were next analyzed to find those harboring one H chain and two different L chains (either two κ, two λ, or one κ and one λ chains). Surprisingly, the frequency of κ^+^λ^+^ B cells measured by single cell V(D)J-seq, and of dual-L chain B cells in general, was similar in SLE and control samples (**Figure 5B** and **Supplementary Figure S4A**). Thus, while the frequencies of κ^+^λ^+^ B cells measured by single cell V(D)J-seq and flow cytometry were comparable for the control samples, they were different for the SLE samples (**Supplementary Figure S4B**). The low detection of κ^+^λ^+^ B cells by single cell V(D)J-seq analysis did not appear to be related to inefficient capture by the 10X Genomics platform, because we observed a strong correlation between the frequencies of Ig switched (IgM^–^IgD^–^) B cells measured by single cell V(D)J-seq and flow cytometry (**Figure 5C**).

The VH usage of B cell clonotypes harboring two different light chains was generally similar with that of clonotypes harboring only one light chain, as shown for healthy controls (**Supplementary Figure S4C**). CDR3 amino acid length, a parameter that correlates with self-reactivity, was also comparable between κ^+^ and κ^+^λ^+^ clonotypes, although it was slightly shorter in clonotypes from SLE-H patients relative to healthy controls (**Figure 5D**). Interestingly, κ^+^λ^+^ clonotypes displayed a somewhat skewed Jκ usage (**Figure 5E**), with lower frequency of Jκ1 (difference significant only in control sequences) and higher frequency of Jκ5 (difference significant only is SLE sequences).

In summary, single cell V(D)J-seq analysis indicates that κ^+^λ^+^ cells are about 2% of all B cells in both SLE patients and healthy controls and that they have skewed usage of Jκ genes.

### Correlation Between B_2R_ Cells and Sera VH4-34 Antibodies

About 55-70% of SLE patients have been described to display elevated serum titers of VH4-34 antibodies (13, 14). Antibodies harboring the VH4-34 chain are often autoreactive, and a subset (especially VH4-34 of IgM class (13, 28)) reacts with a B cell-specific epitope and, thus, decorates B cells (14, 29). Hence, we hypothesized that the difference in κ^+^λ^+^ frequencies observed by flow cytometry and single cell V(D)J-seq in SLE-H patients might be due to the presence of VH4-34 autoantibodies that react with B cell antigens.

**Figure 6:**
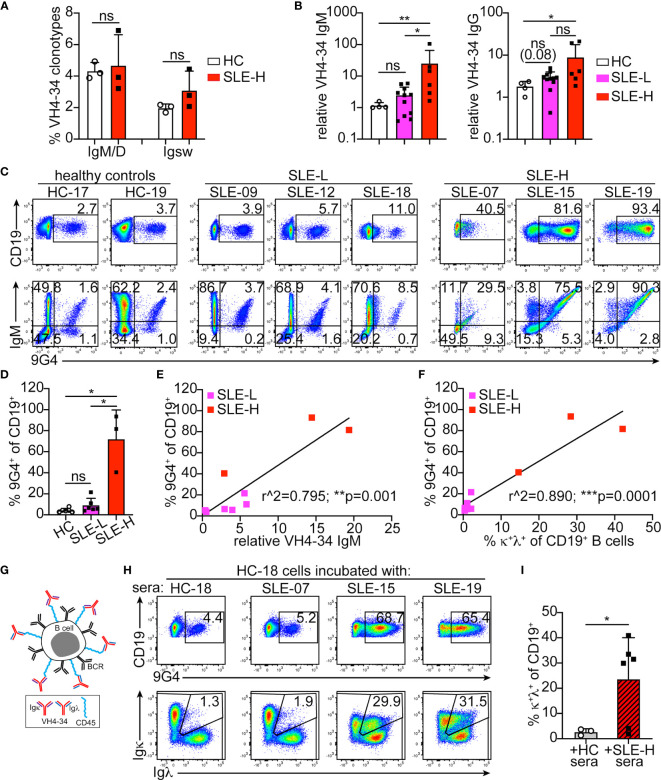
Analysis of VH4-34 B cells and antibodies. **(A)** Frequency of VH4-34-bearing clonotypes among all unswitched (IgM/D) and switched (Igsw) clones obtained by 10X Genomics V(D)J sequencing in three healthy controls (HC) and three SLE-H subjects. **(B)** Relative titers of VH4-34 IgM (left) and VH4-34 IgG (right) in sera of healthy control (HC, N=4), SLE-L (N=11) and SLE-H (N=6) subjects. Titers are shown on logarithmic scale and relative to one of the HC samples. **(C)** Representative flow cytometric analyses of 9G4 (anti-VH4-34) staining vs CD19 (top) and IgM (bottom) on CD19^+^ gated B cells from healthy and SLE subjects. **(D)** Frequency of 9G4^+^ B cells measured by flow cytometry in healthy (N=6), SLE-L (N=6) and SLE-H (N=3) subjects over 4 independent experiments. **(E, F)** Scatter plot analyses of the percentage of 9G4^+^ B cells (y-axis) relative to either (on x-axis) VH4-34 IgM sera titers **(E)** or the frequency of κ^+^λ^+^ B cells **(F)** in SLE patients. N=9, 6 SLE-L and 3 SLE-H. Data were analyzed by simple linear regression. **(G)** Schematic modeling the decoration of B cells by VH4-34, κ or λ, antibodies. **(H)** Flow cytometric analyses of CD19 vs 9G4 (top), and Igκ vs Igλ (bottom), on CD19^+^ gated B cells from healthy control HC-18 that were incubated for 30 min at 4°C with undiluted sera from the same healthy subject or from three SLE-H individuals, as indicated. Data are from one of two independent experiments (data from second experiment are in **Supplementary Figure S5D**). **(I)** Frequency of κ^+^λ^+^ cells measured by flow cytometry within the CD19^+^ B cell populations of HC PBMCs incubated with sera from either HC or SLE-H subjects. N=3 for HC cells+HC sera; N=6 for HC cells+SLE-H sera; analyzed over 2 independent experiments. In all bar graphs, each symbol is an individual, bars represent mean+SD, and statistical analyses were performed with Mann-Whitney *U* tests. *p ≤ 0.05; **p < 0.01; ***p < 0.001; ns, not significant.

Based on single cell V(D)J-seq analysis, VH4-34 usage was similar (about 5%) in the healthy controls and SLE-H patients examined (**Figure 6A**). However, when 9G4 monoclonal antibodies (30) were used to measure sera VH4-34 immunoglobulins, we found elevated VH4-34 IgM titers in 60% (11 of 18) of the SLE subjects and, particularly, in those with high κ^+^λ^+^ B cells (**Figure 6B**). VH4-34 IgG titers were also elevated in 44% of the patients and was particularly high in two SLE-H subjects (**Figure 6B**).

To determine the proportion of VH4-34^+^ B cells, we stained PBMCs from a subset of healthy controls and SLE patients with the 9G4 antibody (**Figure 6C**). 9G4^+^ B cells were observed at similar frequencies in the healthy controls and SLE-L patients examined, but were markedly elevated (40%-93% of all B cells) in the SLE-H patients (**Figures 6C, D**). In all individuals analyzed, most 9G4^+^ B cells were IgM^+^ (**Figure 6C**). 9G4^+^IgG^+^ B cells were almost absent in SLE-L and healthy subjects, but were more prevalent in SLE-H subjects (**Supplementary Figure S5A**). There was also a strong correlation between the frequencies of 9G4^+^ B cells and the relative titers of VH4-34 IgM in their sera (**Figure 6E**), but not with VH4-34 IgG (**Supplementary Figure S5B**). An even stronger correlation was observed between the frequencies of 9G4^+^ B cells and κ^+^λ^+^ B cells (**Figure 6F**). In fact, κ^+^λ^+^ cells were increased only in the 9G4^+^ B cell population (**Supplementary Figure S5C**). These data suggest that B cells of SLE-H patients are decorated with VH4-34, mostly of IgM class (**Figure 6G**). The VH4-34 IgM/D sequences from SLE patients did not show any distinctive feature, such as oligoclonal expansion (**Supplementary Table S4**). VH4-34 IgM/D clonotypes from two of the three SLE patients were 90-100% κ^+^ (**Supplementary Table S4**), but the low numbers of clonotype sequences recovered from these samples did not allow to make meaningful conclusions.

To test the ability of the SLE-H sera antibodies to bind B cells, we incubated PBMCs from two healthy controls for 30 minutes on ice (as in (14)) with either the sera from the same individual or another healthy subject, or with sera from the six SLE-H patients. Incubation with sera from four of the six SLE-H patients, but not with sera from HC subjects, significantly increased 9G4-binding and the frequency of B cells falling within the κ/λ double-positive gate (**Figure 6H, I** and **Supplementary Figure S5D**). The two SLE-H patients whose sera did not lead to higher 9G4^+^ and κ^+^λ^+^ B cells were those with the lowest VH4-34 IgM titers (1.6-2.9, relative to 5.2-105.3 in the other SLE-H patients).

In summary, the great majority of κ^+^λ^+^ B cells in SLE patients with high frequency of B_2R_ cells are B cells decorated with VH4-34 autoantibodies and not cells co-expressing two Ig light chains and antibodies.

## Discussion

Allelically and isotypically-included B cells co-expressing two different BCRs and antibodies have been previously identified in healthy mice and humans. Based on the observations that these dual-antibody B cells (B_2R_ cells) are both more frequent and activated in lupus-like MRL and MRL/*lpr* mice, but not necessarily in other lupus-like mouse models, we hypothesized that B_2R_ cells might be expanded in a subset of SLE patients. Here we show that about one fourth of a Colorado SLE patient cohort, and particularly those patients with higher proportion of naïve activated B cells and ABCs and a lower proportion of BNDs, have an apparent higher frequency of κ^+^λ^+^ B_2R_ cells in their blood. However, by performing single cell V(D)J sequencing analysis, our study unequivocally demonstrates that the large majority of κ^+^λ^+^ B cells observed in these SLE patients do not truly express two different light chains, but instead are decorated by VH4-34 autoantibodies (with κ or λ) that bind B cell-specific surface antigens. Based on single cell V(D)J-seq analyses, the frequency of all B_2R_ cells in the SLE patients examined is about 5%, similar in range to healthy controls. Results from our studies indicate that flow cytometric detection of high numbers of κ^+^λ^+^ B cells in blood identifies SLE patients with elevated titers of B cell-specific VH4-34 autoantibodies and other B cell abnormalities.

Studies in mice have led to the conclusion that B_2R_ B cells generally arise during central tolerance as the result of receptor editing, and that they co-express autoreactive and nonautoreactive antibodies [reviewed in (2)]. Indeed, we have previously observed (by both flow cytometric and hybridoma analyses) elevated frequencies of dual-κ B cells in the lupus-like model MRL and the disease accelerated version MRL/*lpr* (8), where these cells further expand and become activated in response to TLR and IFNR signaling (9). Such B_2R_ B cell expansion, in contrast, was not found in another spontaneous lupus mouse strain (10). These previous observations provided the impetus for the studies described herein. A goal of our follow up studies was to clarify the mechanisms supporting the enhanced differentiation of dual-κ B cells into GC B cells in MRL/*lpr* mice. It has been previously shown that type I and type II IFNs can enhance the ability of TLR7 signaling (and possibly also that of TLR9) to promote GC B cell formation (31). We show here that while the synergistic effect of IFNα with R848 is similar in single-κ and dual-κ MRL/*lpr* B cells, IFNγ synergizes more potently with R848 in dual-κ B cells, leading to enhanced differentiation of B_2R_ cells into GC B cells. This difference is also consistent with the autoreactivity of dual-κ B cells and the reported ability of BCR signaling to further synergize with TLR7 and IFNγ receptor signaling in the upregulation of GC markers (31).

Allelically-included B cells (specifically κ^+^λ^+^) have been described in humans at frequencies similar to those observed in healthy mice (3, 4). Thus, a major goal of our studies was to investigate frequencies and characteristics of κ^+^λ^+^ B cells in SLE subjects, positing they would be elevated (and more activated) in a fraction of patients. This is indeed what we observed by flow cytometry and microscopy in one fourth of our SLE patients and one tenth of ALPS samples. This observation, moreover, was consistent with a previously published study in which half of a UK SLE patient cohort displayed a higher frequency of κ^+^λ^+^ B cells (11). However, using single cell V(D)J-seq analysis, we clearly show here that κ^+^λ^+^ B cells, and B_2R_ cells in general, are not elevated in our SLE patients with apparent high κ^+^λ^+^ B cells. Our findings do not exclude that few SLE patients harboring a *bona fide* expansion of isotypically-included B cells exist in the population at large. However, our data demonstrate that high numbers of B_2R_ cells is not a common characteristic of systemic autoimmunity and/or lymphoproliferation in humans, and that a more realistic measure of allelic/isotypic exclusion in B cells requires single cells V(D)J-seq analysis in addition to flow cytometry. Some genuine dual light chain B cells (about 5% of all B cells) were identified by single cell V(D)J-seq in both patients and controls and the only difference we observed in real κ^+^λ^+^ B cells was a Jκ usage skewed toward distal Jκs, a characteristic compatible with increased rounds of receptor editing.

The discrepancy between the single cell V(D)J-seq and the flow cytometry (and microscopy) data led to realization that the large number of κ^+^λ^+^ B cells measured by flow cytometry in the SLE-H patient subgroup was due to the decoration of B cells by VH4-34 autoantibodies, and not to isotypic inclusion. The VH4-34 heavy chain has been described to contain a germline-encoded hydrophobic patch that mediates binding to I/i carbohydrates expressed on the surface of red blood cells and some B cells (13, 14, 28–30). The ability of VH4-34 to bind B cells is influenced by CDR3 charges and the type of L chain, such that only about 60% of VH4-34 IgM antibodies are actually able to bind B cells (28). Some VH4-34 antibodies also bind DNA and other lupus-related antigens (28), but while the autoreactive VH4-34 B cells undergo peripheral tolerance in healthy individuals, they differentiate into antibody-secreting cells in 50-70% of SLE patients (13, 14, 25, 32–34). We detected elevated VH4-34 of IgM and IgG isotypes in 60% and 44% of our SLE subjects, respectively. This frequency is in line with published reports, although IgG is more common than IgM in other cohorts (13, 14). Elevated VH4-34 titers were observed more frequently in SLE-H patients (80% of this group) than in SLE-L (54%), and these titers were particularly high in half of the SLE-H subjects. In correlation with VH4-34 titers, sera from SLE patients have been previously demonstrated to bind and decorate B cells, causing B cells to appear VH4-34 (9G4)-positive (13, 14). In line with these reports, our SLE patients with the highest VH4-34 titers also showed the highest frequencies of 9G4^+^ B cells. Moreover, serum from these patients was able to decorate B cells from healthy controls leading to a significant apparent increase of 9G4^+^ B cells, and similar to what observed by others (14).

Extending previous findings, our data show that decoration of B cells by VH4-34 antibodies results in an increased apparent frequency of κ^+^λ^+^ B cells. Supporting this conclusion, the abnormal frequency of κ^+^λ^+^ cells was only present in the 9G4^+^ (and not the 9G4^–^) B cell subset of SLE-H patients. Moreover, when B cells from healthy controls were incubated with sera from SLE-H patients, this caused a rise in the frequency of observed κ^+^λ^+^ B cells, paralleling the increase in 9G4^+^ B cells. Although a positive correlation exists between frequency of 9G4^+^ B cells and κ^+^λ^+^ B cells in the SLE-H patients, the frequency of 9G4^+^ B cells was higher than that of κ^+^λ^+^ B cells in this cohort. However, κ^+^λ^+^ B cells may have been possibly undercounted depending on how much VH4-34 antibody was bound and whether this antibody was predominantly kappa or lambda. Indeed, the κ^+^λ^+^ B cells in SLE-H patients were either κ^high^λ^+^ or κ^+^λ^high^. Given VH4-34 antibodies that bind B cells are not skewed for L chain isotype (14), our observation likely results from whether the decorated B cells *per se* expressed Igκ or Igλ BCRs. In the experiments in which healthy controls B cells were incubated with heterologous SLE-H sera, the frequency of κ^+^λ^+^ B cells was lower than that observed among the original SLE-H B cells. This perhaps is because the 30’ incubation with sera was insufficient to achieve the level of VH4-34 decoration that B cells from SLE-H patients attained *in vivo*, while circulating in the VH4-34-enriched blood. The reduced B cell decoration achieved *in vitro* could also stem from the fact that it was performed in ice, while the original antibodies bound B cells *in vivo* at 37°C. Based on the distribution of κ^+^λ^+^ B cells in patients, and the profile of B cells decorated by 9G4, the VH4-34 autoantibodies from SLE-H patients bound MZ-like B cells and all CD27^–^ B cell subset, excluding BNDs. This differential ability of VH4-34 to decorate B cell subsets is generally in line with what previously described (14).

Double negative (DN), activated naïve (aNAV), age-associated B cell (ABC), and B cells that are naïve IgD (BND) B cell subsets are relevant to autoimmunity due to their high proportion of clones expressing autoreactive antibodies (21, 23, 25, 35). DN, aNAV, and ABC B cells are precursors of antibody-secreting cells (21, 25), while BNDs are anergic B cells (23, 24). Half or more of the SLE patients with apparent high κ^+^λ^+^ B cells harbored higher frequencies of DN, aNAV, and ABC B cells and a lower frequency of BNDs, when compared to most SLE subjects with normal B_2R_ cells. These observations are generally in line with those established by others in relation to VH4-34 (9G4) titers (20), and indicate a propensity for autoreactive B cells to break tolerance and differentiate into autoantibody-secreting cells in these patients. In large patient cohorts, high frequencies of DNs and ABCs (which belong to the DN2 subset) have been described to occur in about 20% of subjects, on average (20, 21). These frequencies increase significantly with disease severity, such that most severe patients (with a SLEDAI score ≥8) exhibit high proportion of ABC and DN2 B cells, correlating also with higher incidence of nephritis and anti-RNA antibodies (20–22). In our cohort, higher frequencies of DN2 and/or ABCs were observed in 33% and in 50% of SLE-L and SLE-H patients, respectively. Moreover, the SLEDAI score of SLE-H patients trended to be higher, although this difference was not significant. However, the proportion of patients with anti-Smith antibodies or with renal disease was not particularly different in the two groups (42% of SLE-L and 33% of SLE-H). Most likely, the size of our SLE cohort, and the fact that most of the patients had a SLEDAI score ≤6, precludes detecting differences in disease characteristics like those described in larger cohorts in relation to VH4-34 antibodies and DN2/ABC B cells (20–22).

In summary, our study demonstrates that SLE patients as well as individuals with inactivating mutations in the *FAS* gene generally harbor normal frequencies of B_2R_ cells, thus indicating the process of Ig allelic/isotypic exclusion is not perturbed by a systemic autoimmune or lymphoproliferative disorder. Our study also demonstrates that an heightened detection of κ^+^λ^+^ B cells by flow cytometry is able to identify patients with abnormal distribution of autoimmune-relevant B cell subsets as well as high B cell-reactive VH4-34 autoantibodies.

## Data Availability Statement

The VDJ sequencing dataset presented in this study can be found in the following online repository and accession number: https://www.ncbi.nlm.nih.gov/geo/query/acc.cgi?acc=GSE183051. All the other original datasets are included in the article/supplementary material. Further inquiries can be directed to the corresponding author.

## Ethics Statement

The studies involving human participants were reviewed and approved by University of Colorado Institutional Review Board. The patients/participants provided their written informed consent to participate in this study. The animal study was reviewed and approved by University of Colorado Anschutz Medical Campus Institutional Animal Care and Use Committee.

## Author Contributions

RP conceived and designed the overall study. RT provided significant input to the design of the study and interpretation of the data. JP, ST, AS, MK, JL, JR, and AM conducted experiments and data acquisition. ST, AS, and JL designed and performed some of the analyses. RS performed the initial V(D)J-seq data curation. SB provided clinical oversight for the recruitment of most SLE patients. JT provided clinical oversight for the recruitment of some patients. RP and JP consented and enrolled all human subjects. SB collected and recorded all clinical data and calculated all disease activity scores. VR contributed essential samples and related clinical data. RP analyzed all experimental data, generated all figures, tables and graphs, and wrote the manuscript. All authors provided comments and revisions to the manuscript draft. JP, SB, ST, and AS share first co-authorship in the order by which they individually contributed to data presented in figures and tables. All authors contributed to the article and approved the submitted version.

## Funding

This work was supported by the National Institute of Health R01 grants AI052310 and AI124474 to RP and AI136534 to RT, and K24 grant AI078004 to SB. It was also partly supported by the NIH/NCATS Colorado CTSA Grant UL1 TR002535, and the University of Colorado Cancer Center’s Genomics Shared Resource funded by NCI grant P30CA046934. ST, AS, and RS were partly supported by the T32 AI074491 grant.

## Conflict of Interest

JT receives royalties from Alexion Pharmaceuticals, Inc. and is a consultant for Q32 Bio, Inc., a company developing complement inhibitors. He also holds stock and will receive royalty income from Q32 Bio, Inc.

The remaining authors declare that the research was conducted in the absence of any commercial or financial relationships that could be construed as a potential conflict of interest.

## Publisher’s Note

All claims expressed in this article are solely those of the authors and do not necessarily represent those of their affiliated organizations, or those of the publisher, the editors and the reviewers. Any product that may be evaluated in this article, or claim that may be made by its manufacturer, is not guaranteed or endorsed by the publisher.
