# Supplementary material for: Elevated Detection of Dual Antibody B Cells Identifies Lupus Patients With B Cell-Reactive VH4-34 Autoantibodies
Source: Front Immunol. 2022 Feb 4;13:795209. doi: 10.3389/fimmu.2022.795209 (PMC8854503; doi:10.3389/fimmu.2022.795209)
Supplement: Supplementary file 1 [file DataSheet_1.pdf]

Supplementary Material

Elevated detection of dual antibody B cells identifies lupus patients with B cell-reactive VH4-34 autoantibodies

Jacob N. Peterson, Susan A. Boackle, Sophina H. Taitano, Allison Sang, Julie Lang, Margot Kelly, Jeremy T. Rahkola, Anjelica M. Miranda, Ryan M. Sheridan, Joshua M. Thurman, V. Koneti Rao, Raul M. Torres, and Roberta Pelanda

Content: Figures S1-S5, Tables S1-S4

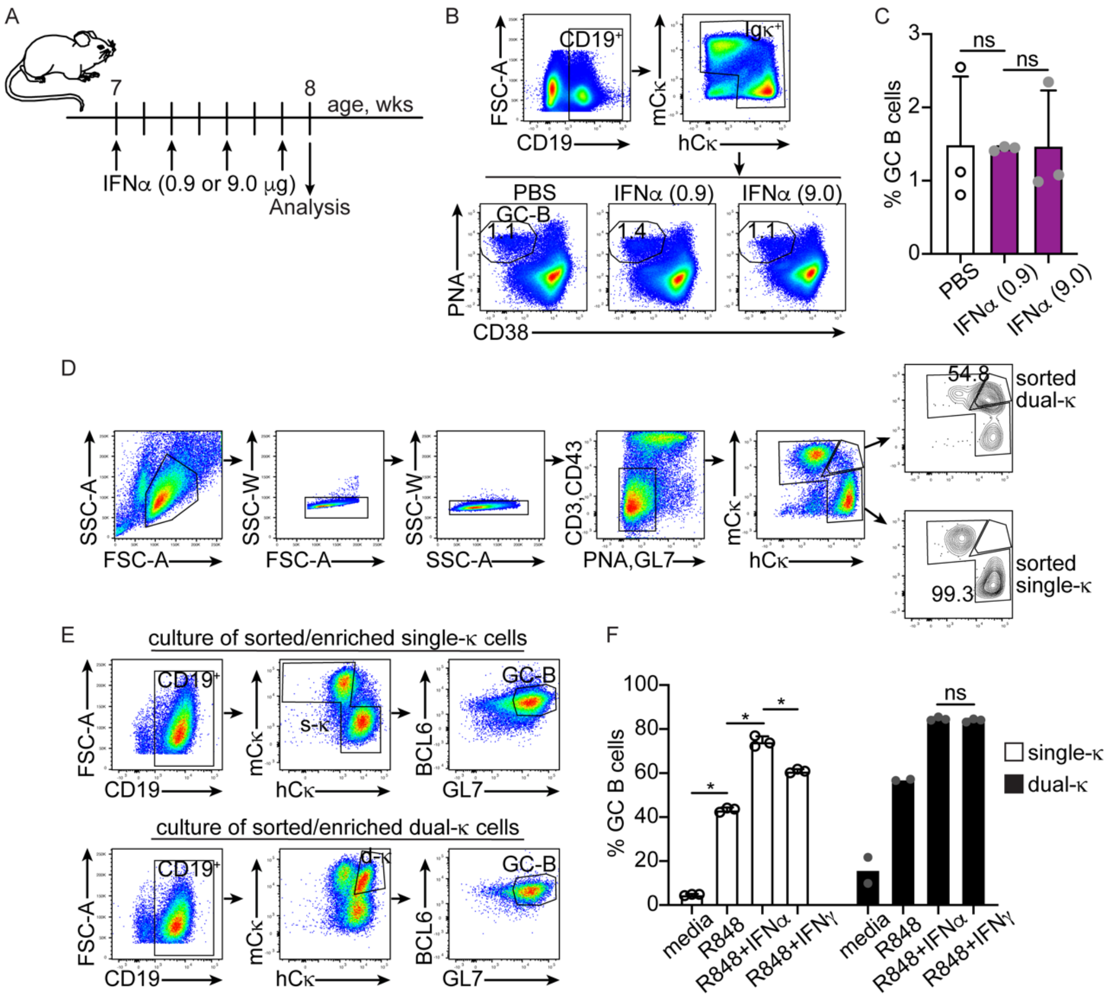

Figure S1: IFNα and IFNγ synergize with TLR7 to promote the differentiation of single-κ and dual-κ MRL/*lpr* B cells into GC B cells.

A) Schematics for the *in vivo* treatment of 7 weeks old MRL/*lpr* mice with PBS or IFNα at two different doses. B) Flow cytometric gating strategy of spleen B cells from MRL/*lpr* mice injected with either PBS or IFNα to measure the frequency of PNA^{high}CD38^{low} GC B cells within all κ^{+} B cells. C) Frequency of PNA^{high}CD38^{low} GC B cells within all κ^{+} B cells from MRL/*lpr* mice treated with either PBS or IFNα. N=3 per group, analyzed over one experiment. D) Fluorescence activated cell sorting of CD43$^{–}$CD3$^{–}$PNA$^{–}$GL7$^{–}$ single-κ and dual-κ (non-GC) B cells from a combined sample of spleen B cells isolated from a total of 12 MRL/*lpr* mice (11-16 weeks of age). The contour plots show the degree of purity of the sorted populations. E) Representative flow cytometric analysis and gating of the enriched single-κ and dual-κ MRL/*lpr* B cells, sorted as shown in (D), after 60 hours of culture in media alone or with R848, R848+IFNα, or R848+IFNγ. The cells were serially gated to measure BCL6^{+}GL7^{high} GC B cells within the single-κ (s-κ) and dual-κ (d-κ) B cell populations. F) Frequencies of BCL6^{+}GL7^{high} GC B cells within the sorted single-κ and dual-κ MRL/*lpr* B cells cultured as described in (E). N=1 biological sample (mix of 12 mice) cultured in 3 replica wells per condition (except N=2 wells for dual-κ cultured in media alone or with R848), analyzed in one experiment. Data in bar graphs are shown as mean+SD. Statistical analysis was performed with a one-tailed Mann-Whitney *U* test. *p≤0.05; ns=not significant.

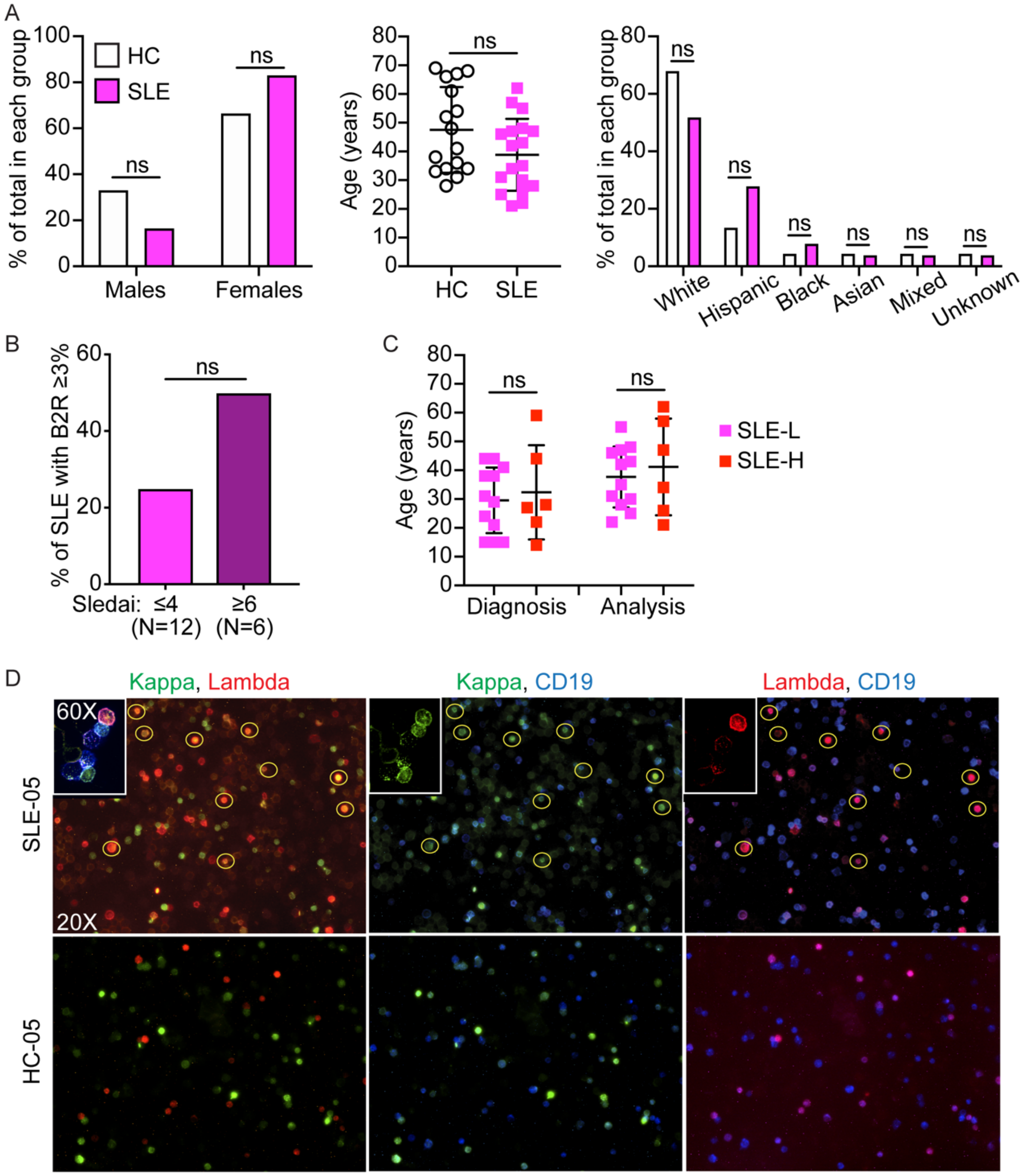

Figure S2: Characteristics of human subjects, and cytospin analysis of $\kappa^+\lambda^+$ B cells.

A) Sex, age (with mean±SD), race and ethnicity distribution of SLE patients and healthy controls. N=18 for both groups. Statistical analysis was performed by either two-tailed Fisher's exact test (sex and race) or t-test (age); ns=not significant. B) Percentage of SLE patients with B_{2R} cells ≥3% within groups with SLEDAI scores ≤4 or ≥6. Statistical analysis was performed by two-tailed Fisher's exact test. C) Age (with mean±SD) of SLE patients at diagnosis and at time of analysis in groups with low B_{2R} cells (SLE-L) and high B_{2R} cells (SLE-H). Statistical analysis was performed by two-tailed Mann-Whitney *U* test. D) Fluorescent microscopy analysis of PBMCs spun on cytospin slides from one SLE patient (SLE-05) and one healthy control (HC-05). The slides were stained for Igκ (green), Igλ (red), and CD19 (blue), and analyzed at 20X magnification (or 60X in insets). The yellow circles highlight cells positive for both κ and λ light chains.

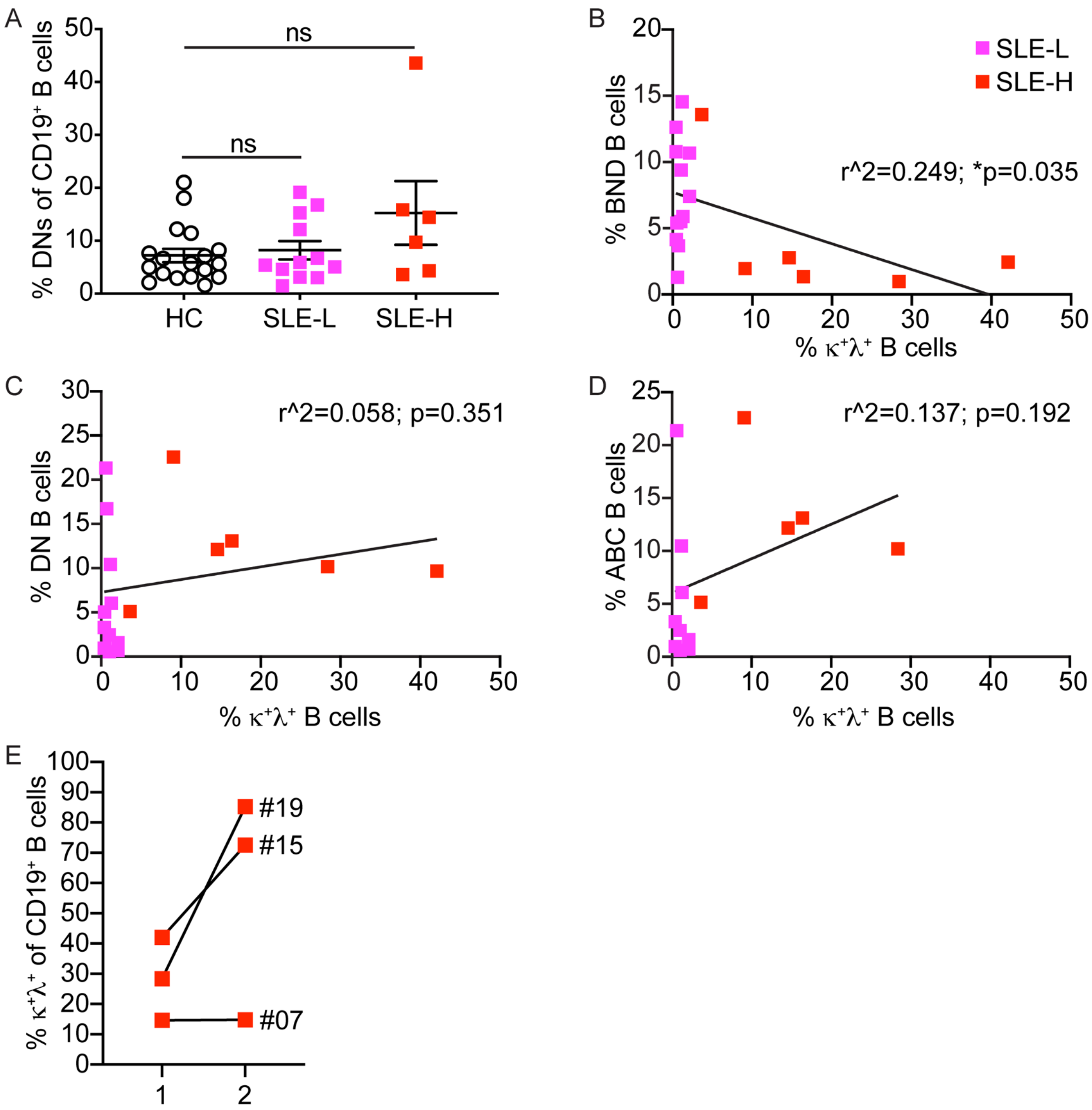

Figure S3: Analysis of B cell subsets and their relationship.

A) Frequency of $CD27^{-}IgD^{-}$ DN cells within the $CD19^{+}$ B cell population of healthy controls (HC, N=18), and SLE patients with low B_{2R} (SLE-L, N=12) or high B_{2R} (SLE-H, N=6) cells. DN cells were gated as shown in Figure 3A. Each symbol is a subject, and bars represent mean±SD. Statistical analysis was performed with a Mann-Whitney *U* test; ns=not significant. B-D) Scatter plot analyses of the percentage of $\kappa^{+}\lambda^{+}$ B cells (x-axis) relative to the percentage (on y-axis) of BND (B), DN (C), or ABC (D) B cells within the $CD19^{+}$ cell population of SLE-H patients. Data were analyzed by Simple linear regression. E) Frequency of $\kappa^{+}\lambda^{+}$ cells within the blood over time. Analyses were performed in two blood samples obtained 6 months to 2 years apart from SLE-H patients # 07, 15, and 19.

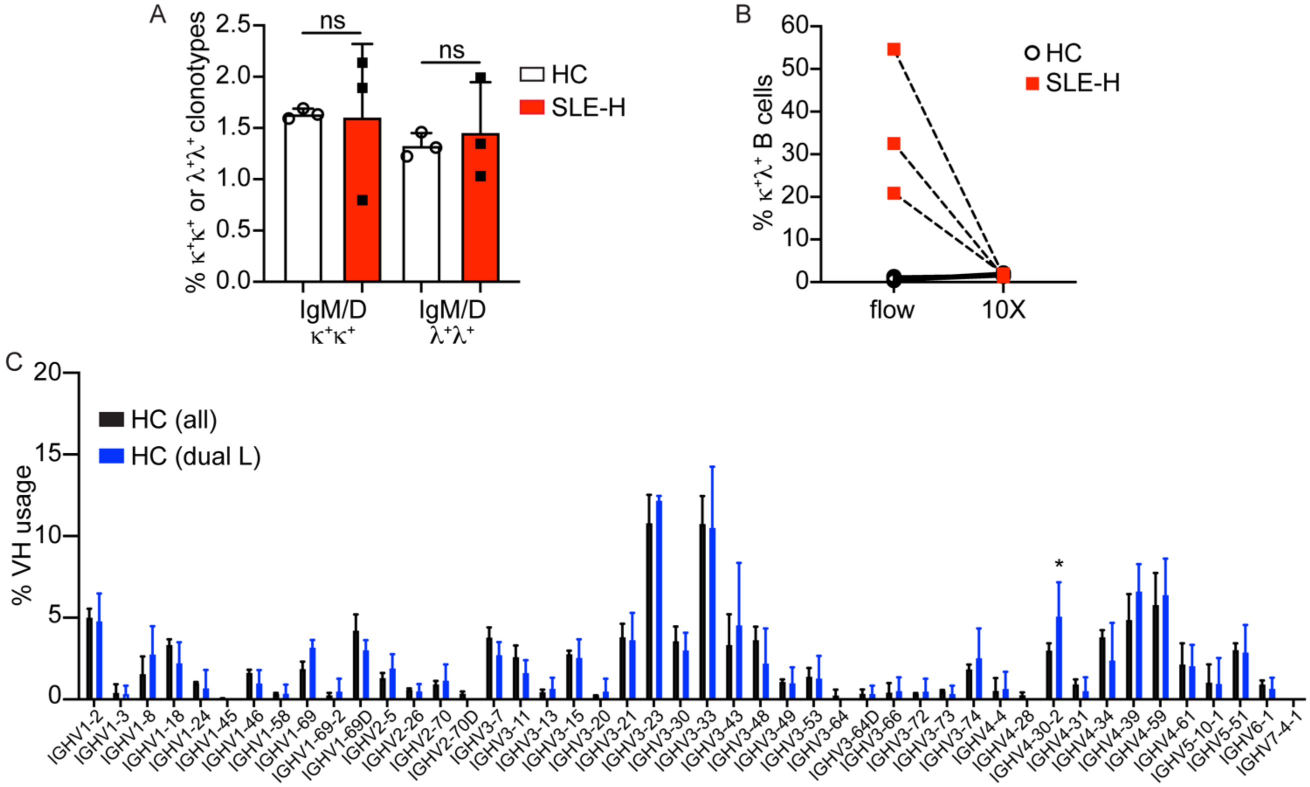

Figure S4: Single cell V(D)J-seq analysis of human B cells.

A) Percentage of unswitched (IgM/D) B cell clonotypes bearing one heavy chain and two κ or two λ genes. Symbols represent individual subjects and bars are mean+SD. Statistical analysis was performed with two-tailed Mann-Whitney *U* tests. B) Percentage of $\kappa^+\lambda^+$ IgM/D B cell clonotypes measured by flow cytometry or 10X in the same SLE-H and healthy control (HC) subjects. Difference was significant only for SLE subjects (p=0.025) as measured by t-test. C) Average (mean+SD) Ig VH usage among either all clonotypes (2,142-5,418 per sample) or dual-light chain ($\kappa^+\kappa^+$, $\lambda^+\lambda^+$, and $\kappa^+\lambda^+$) clonotypes (72-202 per sample) from three healthy controls (HC). Statistical analysis was performed with two-tailed unpaired t-test. *p≤0.05; n.s.=not significant (differences were not significant when lacking asterisks).

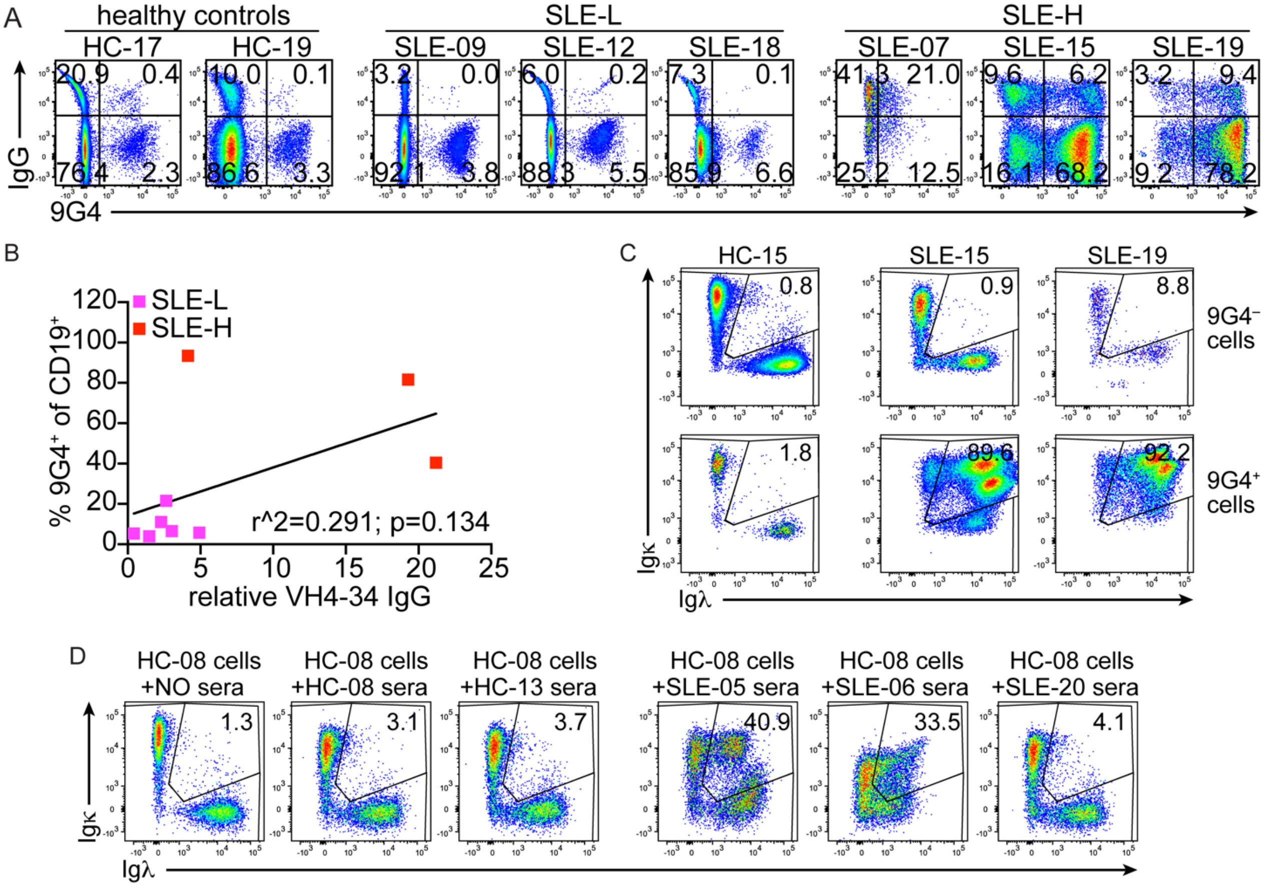

Figure S5: Correlates of VH4-34 secreted antibodies and 9G4⁺ B cells.

A) Representative flow cytometric analyses of 9G4 (anti-VH4-34) staining vs IgG on CD19⁺ gated B cells from healthy and SLE subjects. B) Scatter plot analyses of the percentage of 9G4⁺ B cells (y-axis) relative to VH4-34 IgG relative sera titers (on x-axis) in SLE patients. N=9, 6 SLE-L and 3 SLE-H. Data were analyzed by simple linear regression. C) Representative flow cytometric analyses of Igκ vs Igλ in 9G4⁻ (top) and 9G4⁺ (bottom) CD19⁺ gated B cells. D) Flow cytometric analyses of Igκ vs Igλ on CD19⁺ gated B cells within healthy control HC-08 PBMCs that were incubated for 30 min at 4ºC without or with sera from the same or a different healthy subject, or with sera from SLE-H individuals. All plots are representative of four healthy controls, four SLE-L patients, and three SLE-H patients analyzed over 4 independent analyses.

Table S1: Characteristics of SLE patients and Heathy controls (HC)

*ID #	Sex	**Race& Ethnicity	Age, diagnosis (years)	Age, analysis	Clinical manifestations	***Medications	SLEDAI score	PTPN22 allele	%$\kappa^+\lambda^+$ of $CD19^+$
SLE-01	M	W/NH	15	30	Demyelinating CNS lupus; Arthritis; Lymphopenia; ANA	HCQ; Triamterene-HCTZ	0	R620	1.19
SLE-02	F	AA/NH	31	35	Discoid rash; Photosensitivity; Oral/nasal ulcers; Pericarditis, Pleuritis; Inflammatory myositis; Raynaud's; Lymphopenia; Lymphadenopathy; ANA; anti-Sm, RNP, SSA	HCQ; Aspirin; MM	4	R620	0.72
SLE-03	M	W/NH	38	42	Pericarditis; Arthritis; Psoriasis; Lymphopenia; Eosinophilic esophagitis; partial IgA deficiency; ANA	HCQ; MTX; Folic acid	0	R620	0.5
SLE-04	F	M/H	29	31	Malar rash; Photosensitivity; Pleuritis; Renal disease WHO III; Arthritis; Raynaud's; Slerodactyly; Retinal vasculitis; Lymphopenia; Lymphadenopathy; ANA; anti-dsDNA, Sm	HCQ; Prednisone; Lisinopril	24	R620	1.25
SLE-05	F	W/NH	44	47	Pericarditis; Lymphopenia; ANA; anti-dsDNA, SSA	HCQ; Colchicine	2	R620	16.4
SLE-06	F	W/NH	59	62	Hemolytic anemia; Arthritis; Lymphopenia; Leukopenia; ANA; lupus anticoagulant; anti-dsDNA, cardiolipin, B2GP1	HCQ; Aspirin; Losartan-HCTZ	2	R620	9.07
SLE-07	F	W/H	28	34	Malar rash; Photosensitivity; Pericarditis; Renal disease WHO IV; Arthritis;	Azathioprine; Prednisone; Metformin; Hydralazine	8	R620	14.6

					Lymphopenia; Leukopenia; ANA; anti-dsDNA, Sm, cardiolipin				
SLE-08	F	W/NH	38	55	Pleuritis; Oral/nasal ulcers; Hypothyroidism; breast cancer; Lymphopenia; Leukopenia; Thrombocytopenia; ANA; lupus anticoagulant	HCQ; Aspirin; Cevimeline; Losartan-HCTZ; Meloxicam	2	R620	0.61
SLE-09	M	U/H	15	28	Malar rash; lupus encephalopathy; Renal disease WHO III; Arthritis; Pancarditis; Pancreatitis; Myositis; Lymphopenia; Leukopenia; Thrombocytopenia; ANA; anti-dsDNA, Sm, histone, SSB	HCQ; Lisinopril; Hydralazine; Prednisone; MA	2	R620	0.41
SLE-10	F	W/H	15	22	Malar rash; Discoid rash; Proteinuria, Hematuria; Arthritis; ANA; anti-dsDNA, cardiolipin, B2GP1, PS; PT	None	6	R620	2.09
SLE-12	F	W/H	24	43	Renal disease WHO IV; Arthritis; Leukopenia; ANA; anti-dsDNA	HCQ	2	R620	2.09
SLE-13	F	W/NH	21	25	Malar rash; Renal disease WHO III; ANA; anti-dsDNA, SM, RNP	HCQ; MM; Ditiazem	2	R620	1.0
SLE-15	F	AA/NH	27	57	Photosensitivity; Oral/nasal ulcers; Pericarditis; Arthritis; Congestive heart failure; Lymphopenia; Leukopenia; ANA; anti-cardiolipin	Metoprolol	4	R620	42.1
SLE-16	F	W/NH	41	46	Arthralgia; Raynaud's; Sjogren's;	HCQ; Aspirin	2	R620	0.43

					Lymphopenia; ANA; anti-SSA, SSB				
SLE-18	F	W/NH	44	48	Malar rash; Photosensitivity; Alopecia; Lymphopenia; ANA; anti-dsDNA, Sm	HCQ	2	R620	1.04
SLE-19	F	W/H	14	21	Pleuritis; Pericarditis; Renal disease WHO III; Arthritis; Pulmonary hypertension; ANA; anti-dsDNA; B2GP1	HCQ; Prednisone; MF; aspirin; Ambrisentan; Ivabradine; Digoxin; Omeprazole; Bactrim; Tadalafil	6	R620	28.4
SLE-20	F	W/H	22	26	Discoid rash; Photosensitivity; Oral/nasal ulcers; Pleuritic chest pain; Arthritis; Leukopenia; Thrombocytopenia; ANA	HCQ; Prednisone; MF	6	R620	3.62
SLE-21	F	A/H	44	47	Malar rash; Oral/nasal ulcers; Inflammatory myositis; Raynaud's; Esophageal dysmotility; Lymphopenia; ANA; anti-dsDNA	HCQ	6	R620	0.39
HC-01	M	W/NH	N/A	54	N/A	N/A	N/A	N/A	1.42
HC-02.2	F	AA/NH	N/A	N/A	N/A	N/A	N/A	N/A	0.14
HC-03	M	W/NH	N/A	N/A	N/A	N/A	N/A	N/A	1.12
HC-04	F	N/A	N/A	66	N/A	N/A	N/A	N/A	0.38
HC-05	F	W/NH	N/A	34	N/A	N/A	N/A	N/A	0.46
HC-06	M	W/NH	N/A	69	N/A	N/A	N/A	N/A	0.6
HC-07	F	W/NH	N/A	41	N/A	N/A	N/A	N/A	0.7
HC-08	F	M/H	N/A	34	N/A	N/A	N/A	N/A	0.36
HC-09	M	W/NH	N/A	31	N/A	N/A	N/A	N/A	1.35
HC-11	F	W/NH	N/A	52	N/A	N/A	N/A	N/A	0.71
HC-12	M	W/NH	N/A	67	N/A	N/A	N/A	N/A	0.64
HC-13	F	W/H	N/A	33	N/A	N/A	N/A	N/A	0.25
HC-14.2	F	W/NH	N/A	61	N/A	N/A	N/A	N/A	1.25

HC-15	M	W/NH	N/A	36	N/A	N/A	N/A	N/A	0.38
HC-17	F	W/NH	N/A	48	N/A	N/A	N/A	N/A	0.91
HC-18	F	W/H	N/A	28	N/A	N/A	N/A	N/A	0.23
HC-19	F	W/NH	N/A	68	N/A	N/A	N/A	N/A	1.76
HC-21	F	A/NH	N/A	38	N/A	N/A	N/A	N/A	0.41

*SLE patients in red are those with abnormally high frequency of $\kappa^+\lambda^+$ B cells and designated SLE-H in the study

**W=White; AA=African American or Black; A=Asian; M=Mixed race; U=Unknown race;

NH=Not Hispanic; H=Hispanic

***HCQ=Hydroxychloquine; MTX=Methotrexate; MM=Mycophenolic mofetil; MA=Mycophenolic acid

Table S2: Characteristics of ALPS patients

ID #	Age	Clinical manifestations	%$\kappa^+\lambda^+$ of $CD20^+$
243.1	3	thrombocytopenia, neutropenia	2.59
243.2	4	anemia, thrombocytopenia	12.03
243.3	10	neutropenia	1.55
243.4	13	uveitis	2.45
243.5	18	thrombocytopenia	1.70
243.6	6	neutropenia, thrombocytopenia, anemia	2.86
243.7	16	uveititis, nephritis	1.17
243.8	19	anemia, thrombocytopenia	0.36
243.9	8	neutropenia, thrombocytopenia, anemia	0.35
243.10	24	anemia	0.49

Table S3: Efficiency of B cell preparation and capture for single cell RNA-seq analyses

*ID #	**% live cells	**% CD19^{+} of live cells	Total cell #	B cell # (VDJ barcodes)	% B cells of total cells	Unique B cell clonotypes	***B cell clonotypes with H and L
SLE-07	88.6	30.9	3,388	1,239	36.6	1,168	908
SLE-15	93.8	13.1	6,543	951	14.5	887	831
SLE-19	91.7	76.7	5,143	4,510	87.7	4,153	4,005
HC-07	78.1	73.2	4,321	3,439	79.6	3,376	3,310
HC-15	95.6	95.5	5,554	5,431	97.8	5,418	5,316
HC-19	65.8	90.7	2,445	2,189	89.6	2,142	1,916

*HC-07, HC-19, SLE-07, SLE-19 were processed by 10X Genomics and sequenced independently from HC-15 and SLE15 samples.

**% of live cells and of CD19^{+} cells were measured by flow cytometry after magnetic depletion of dead cells and non-B cells.

***Clonotypes are B cells with unique V(D)J sequence heavy and light chain combinations.

Supplemental Table 4: VH4-34 B cell clonotypes from SLE patients and Heathy controls (HC)

Sample	clonotype_id	igh_cdr3s	igh_v–genes	igh_j	igh_c	igl_v–genes	igl_j	igl_c	igk_v_genes	igk_j	igk_c
**SLE-07**	clonotype630	CARGSPGSSWPRGYW	IGHV4-34	IGHJ4	IGHM				IGKV1-5	IGKJ2	IGKC
	clonotype12	CARGTGYYGETGRDYYYYYGMDVW	IGHV4-34	IGHJ6	IGHD				IGKV1-9	IGKJ2	IGKC
	clonotype1161	CARGATKGYCTNGVCENGPPFDYW	IGHV4-34	IGHJ4	IGHM				IGKV1-27	IGKJ1	IGKC
	clonotype1009	CARGIGGSGPW	IGHV4-34	IGHJ4	IGHM				IGKV1-39	IGKJ2	IGKC
	clonotype289	CARWRRGTNGVHDYW	IGHV4-34	IGHJ4	IGHD				IGKV1D-39	IGKJ3	IGKC
	clonotype24	CARGRTTVTRGAFDIW	IGHV4-34	IGHJ3	IGHD				IGKV3-20	IGKJ1	IGKC
	clonotype343	CARGWSTTYYYGSGIARW	IGHV4-34	IGHJ4	IGHM				IGKV3-20	IGKJ1	IGKC
	clonotype574	CARGRSQHLYYYYYYMDVW	IGHV4-34	IGHJ6	IGHD				IGKV3D-15	IGKJ4	IGKC
	clonotype583	CARRGLRLGELSSYAPFDYW	IGHV4-34	IGHJ4	IGHM				IGKV4-1	IGKJ2	IGKC

Sample	clonotype_id	igh_cdr3s	igh_v–genes	igh_j	igh_c	igl_v–genes	igl_j	igl_c	igk_v_genes	igk_j	igk_c
**SLE-15**	clonotype258	CAREEDTAMVCAFDIW	IGHV4-34	IGHJ3	IGHM				IGKV1-8	IGKJ3	IGKC
	clonotype807	CARVCIRSSGWYGVIDYW	IGHV4-34	IGHJ4	IGHM				IGKV1-33	IGKJ4	IGKC
	clonotype378	CAGARLGFDYW	IGHV4-34	IGHJ4	IGHM				IGKV1D-13;IGKV2-24	IGKJ5;IGKJ2	IGKC;IGKC
	clonotype886	CASTQYDFWSGYEDYW	IGHV4-34	IGHJ4	IGHM				IGKV1D-17	IGKJ2	IGKC
	clonotype599	CARGPRRIAARRGGWFDPW	IGHV4-34	IGHJ5	IGHM				IGKV1D-33	IGKJ1	IGKC
	clonotype462	CVKNYYDSSGYAFDIW	IGHV4-34	IGHJ3	IGHM				IGKV1D-39	IGKJ3	IGKC
	clonotype115	CARGRYFDWLFFDYW	IGHV4-34	IGHJ4	IGHM				IGKV1D-39	IGKJ1	IGKC
	clonotype729	CARGVLLWFGAIRGNWFDPW	IGHV4-34	IGHJ5	IGHM				IGKV1D-39	IGKJ1	IGKC
	clonotype86	CAREAGIAAAVKRWWFDPW	IGHV4-34	IGHJ5	IGHM				IGKV1D-39	IGKJ1	IGKC
	clonotype659	CARGYDFWSYHMDVW	IGHV4-34	IGHJ6	IGHM				IGKV1D-39	IGKJ1	IGKC
	clonotype193	CARSMIVVVSSYWYFDLW	IGHV4-34	IGHJ2	IGHM				IGKV2-28	IGKJ4	IGKC
	clonotype854	CARGANYYGSGHWFDPW	IGHV4-34	IGHJ5	IGHM				IGKV3-11	IGKJ5	IGKC
	clonotype804	CARGFQSGNGSGIDYW	IGHV4-34	IGHJ4	IGHM				IGKV3-20	IGKJ5	IGKC
	clonotype164	CARAVPEMTTVVDFDYW	IGHV4-34	IGHJ4	IGHM				IGKV3D-15	IGKJ2	IGKC
	clonotype108	CARGIAAAARHYYYYMDVW	IGHV4-34	IGHJ6	IGHM				IGKV3D-20	IGKJ5	IGKC
	clonotype139	CARLGVVVAASIW	IGHV4-34	IGHJ3	IGHM				IGKV4-1	IGKJ1	IGKC
	clonotype263	CARAPSGGSYYNYFDYW	IGHV4-34	IGHJ4	IGHM				IGKV4-1	IGKJ4	IGKC
	clonotype132	CATRAPTGWYFDFW	IGHV4-34	IGHJ2	IGHD	IGLV1-40	IGLJ3	IGLC3			
	clonotype266	CARRDAQTNSGSYFRRNLGHWFDPW	IGHV4-34	IGHJ5	IGHM	IGLV2-11	IGLJ3	IGLC2			

Sample	clonotype_id	igh_cdr3s	igh_v–genes	igh_j	igh_c	igl_v–genes	igl_j	igl_c	igk_v_genes	igk_j	igk_c
**SLE-19**	clonotype3932	CARGVTAAAAVSPNFDYW	IGHV4-34	IGHJ4	IGHM				IGKV1-5	IGKJ4	IGKC
	clonotype1874	CARALSSTSFKYYFDSW	IGHV4-34	IGHJ4	IGHM				IGKV1-5	IGKJ2	IGKC
	clonotype1024	CARGSNTYDYW	IGHV4-34	IGHJ4	IGHM				IGKV1-5	IGKJ1	IGKC
	clonotype660	CARPRYCSGGSCSRALDYW	IGHV4-34	IGHJ4	IGHM				IGKV1-5	IGKJ2	IGKC
	clonotype3856	CARVRYQVGDFLPHGMDVW	IGHV4-34	IGHJ6	IGHM				IGKV1-5	IGKJ1	IGKC
	clonotype28	CARGGGYCSGGSCYSLLDYW	IGHV4-34	IGHJ4	IGHM				IGKV1-6	IGKJ2	IGKC
	clonotype1601	CARGKEAVLRVRGGNSWFDPW	IGHV4-34	IGHJ5	IGHM				IGKV1-8	IGKJ4	IGKC
	clonotype599	CARGQTTVVTYYYYGMDVW	IGHV4-34	IGHJ6	IGHM				IGKV1-8	IGKJ1	IGKC
	clonotype2292	CARDSGGDYQDYW	IGHV4-34	IGHJ4	IGHM				IGKV1-9	IGKJ4	IGKC
	clonotype4116	CARGITMVRGVPYW	IGHV4-34	IGHJ4	IGHM				IGKV1-9	IGKJ5	IGKC
	clonotype2006	CARNSIAAADHYYYYGMDVW	IGHV4-34	IGHJ6	IGHD				IGKV1-9	IGKJ5	IGKC
	clonotype3990	CARGGGITMIVAPPHYYGMDVW	IGHV4-34	IGHJ6	IGHM				IGKV1-9;IGKV2D-40	IGKJ1;IGKJ1	IGKC;IGKC
	clonotype3578	CARVDRTYGSGSSSGMDVW	IGHV4-34	IGHJ6	IGHM				IGKV1-9;IGKV4-1	IGKJ3;IGKJ2	IGKC;IGKC
	clonotype2497	CARSYYDSSGHGWYFDLW	IGHV4-34	IGHJ2	IGHM				IGKV1-12	IGKJ5	IGKC
	clonotype3244	CARLRRGYCSGGSCRVERYFDLW	IGHV4-34	IGHJ2	IGHM				IGKV1-12	IGKJ4	IGKC
	clonotype1749	CASLYGSGSYSNHW	IGHV4-34	IGHJ4	IGHM				IGKV1-16	IGKJ2	IGKC
	clonotype1287	CARVKYSSGWYRGWFDPW	IGHV4-34	IGHJ5	IGHM				IGKV1-16	IGKJ2	IGKC
	clonotype3576	CARGRLIAVLLPRAGEFDYW	IGHV4-34	IGHJ4	IGHM				IGKV1-16;IGKV1-5	IGKJ4;IGKJ4	IGKC;IGKC

	clonotype2587	CARGRGDYW	IGHV4-34	IGHJ4	IGHM				IGKV1-17	IGKJ2	IGKC
	clonotype511	CARGAGVDTAMVPFDYW	IGHV4-34	IGHJ4	IGHM				IGKV1-17	IGKJ2	IGKC
	clonotype682	CARGSRGLRGDYW	IGHV4-34	IGHJ4	IGHM				IGKV1-17	IGKJ2	IGKC
	clonotype3008	CARGGIDIVVVVAANPWYFDYW	IGHV4-34	IGHJ4	IGHM				IGKV1-17	IGKJ4	IGKC
	clonotype1104	CARGGDRNWFDPW	IGHV4-34	IGHJ5	IGHM				IGKV1-17	IGKJ3	IGKC
	clonotype3855	CARFGRDGYGYW	IGHV4-34	IGHJ4	IGHM				IGKV1-17;IGKV2-30	IGKJ3;IGKJ4	IGKC;IGKC
	clonotype3501	CARSPYYDRHFDYW	IGHV4-34	IGHJ4	IGHM				IGKV1-27	IGKJ1	IGKC
	clonotype3130	CARGAHSGYDDYW	IGHV4-34	IGHJ4	IGHM				IGKV1-27	IGKJ1	IGKC
	clonotype3164	CARVRITMVRGSRYFDYW	IGHV4-34	IGHJ4	IGHM				IGKV1-33	IGKJ2	IGKC
	clonotype1165	CARGLLTEDIVAYYYYGVDVW	IGHV4-34	IGHJ6	IGHD				IGKV1-39	IGKJ1	IGKC
	clonotype3159	CARGRFRDYGLRYFDYW	IGHV4-34	IGHJ4	IGHM				IGKV1D-16	IGKJ4	IGKC
	clonotype3572	CARGGPYGGNSGYW	IGHV4-34	IGHJ4	IGHM				IGKV1D-33	IGKJ2	IGKC
	clonotype1064	CATGDSGYVPRW	IGHV4-34	IGHJ4	IGHM				IGKV1D-33	IGKJ4	IGKC
	clonotype1506	CARGPGWFDPW	IGHV4-34	IGHJ5	IGHM				IGKV1D-33	IGKJ4	IGKC
	clonotype1657	CAISSGSKGYFQHW	IGHV4-34	IGHJ1	IGHM				IGKV1D-39	IGKJ2	IGKC
	clonotype3256	CARTSSGYYYDSSGRDAFDIW	IGHV4-34	IGHJ3	IGHM				IGKV1D-39	IGKJ2	IGKC
	clonotype98	CASTGEGQQLAQGYW	IGHV4-34	IGHJ4	IGHM				IGKV1D-39	IGKJ4	IGKC
	clonotype3320	CARSNRGVVTPDYFDYW	IGHV4-34	IGHJ4	IGHD				IGKV1D-39	IGKJ2	IGKC
	clonotype2289	CARGGGADDYGDYRFDYW	IGHV4-34	IGHJ4	IGHM				IGKV1D-39	IGKJ5	IGKC
	clonotype824	CARGGFPGYSYGLDYW	IGHV4-34	IGHJ4	IGHM				IGKV1D-39	IGKJ1	IGKC
	clonotype3308	CARETAVAVNFDYW	IGHV4-34	IGHJ4	IGHM				IGKV1D-39	IGKJ3	IGKC
	clonotype1634	CASIAARRGGSPFDYW	IGHV4-34	IGHJ4	IGHM				IGKV1D-39	IGKJ2	IGKC
	clonotype1757	CARPRYCSGGSCSRALDYW	IGHV4-34	IGHJ4	IGHM				IGKV1D-39	IGKJ5	IGKC
	clonotype1850	CARSLGYCSSTSCSNFDYW	IGHV4-34	IGHJ4	IGHM				IGKV1D-39	IGKJ1	IGKC
	clonotype2590	CARAPRIAAAGTPDW	IGHV4-34	IGHJ4	IGHM				IGKV1D-39	IGKJ4	IGKC
	clonotype3592	CARPTGYSSSWFDYW	IGHV4-34	IGHJ4	IGHM				IGKV1D-39	IGKJ1	IGKC
	clonotype2554	CARDPRLEQQLVRGAFDPW	IGHV4-34	IGHJ5	IGHM				IGKV1D-39	IGKJ1	IGKC
	clonotype3720	CARGRLRSTFRGSTAPAWFDPW	IGHV4-34	IGHJ5	IGHM				IGKV1D-39	IGKJ3	IGKC
	clonotype807	CAREVARCSGGSCYSGVDYYGMDVW	IGHV4-34	IGHJ6	IGHM				IGKV1D-39	IGKJ1	IGKC
	clonotype830	CARFDYGDQPGYYYYGMDVW	IGHV4-34	IGHJ6	IGHM				IGKV1D-39;IGKV2-28	IGKJ4;IGKJ2	IGKC;IGKC
	clonotype3926	CARGIPSRHSSGLDAFDIW	IGHV4-34	IGHJ3	IGHM				IGKV2-28	IGKJ1	IGKC
	clonotype3449	CARVKEPGIAAAAAGIAFDIW	IGHV4-34	IGHJ3	IGHM				IGKV2-28	IGKJ1	IGKC
	clonotype9	CARGVVGEKALYYFDYW	IGHV4-34	IGHJ4	IGHM				IGKV2-28	IGKJ1	IGKC
	clonotype1778	CARRALGIAAAFIW	IGHV4-34	IGHJ3	IGHM				IGKV2-28;IGKV2-30	IGKJ2;IGKJ4	IGKC;IGKC
	clonotype979	CAREEVGATVYW	IGHV4-34	IGHJ4	IGHM				IGKV2-30	IGKJ1	IGKC
	clonotype5	CARGRYFDYW	IGHV4-34	IGHJ4	IGHM				IGKV2D-29	IGKJ4	IGKC
	clonotype2137	CARWDGVRGNSRTPFDYW	IGHV4-34	IGHJ4	IGHM				IGKV2D-29	IGKJ2	IGKC
	clonotype143	CARGPTIGLDPW	IGHV4-34	IGHJ5	IGHD				IGKV2D-29	IGKJ2	IGKC
	clonotype371	CARGVFPGPYFDLW	IGHV4-34	IGHJ2	IGHM				IGKV3-11	IGKJ5	IGKC
	clonotype741	CARDNAVHGGAFDIW	IGHV4-34	IGHJ3	IGHM				IGKV3-11	IGKJ1	IGKC
	clonotype2760	CARGPEAAAGTQYYFDYW	IGHV4-34	IGHJ4	IGHM				IGKV3-11	IGKJ4	IGKC
	clonotype2010	CASRFTIWRGADYW	IGHV4-34	IGHJ4	IGHM				IGKV3-11	IGKJ3	IGKC
	clonotype3518	CARGRINTFGGVFDYW	IGHV4-34	IGHJ4	IGHM				IGKV3-11	IGKJ3	IGKC
	clonotype749	CARGLLDTAMVFPYYFDYW	IGHV4-34	IGHJ4	IGHM				IGKV3-11	IGKJ4	IGKC
	clonotype2897	CASRQPSSSWYGNWFDPW	IGHV4-34	IGHJ5	IGHM				IGKV3-11	IGKJ5	IGKC
	clonotype4033	CARVLAAAETGYYYGMDVW	IGHV4-34	IGHJ6	IGHD				IGKV3-11	IGKJ2	IGKC
	clonotype2274	CARVAGTGPVVIDYW	IGHV4-34	IGHJ4	IGHD				IGKV3-11;IGKV3-20	IGKJ4;IGKJ4	IGKC;IGKC
	clonotype3012	CARGVPLGDCIGGSCYSAFDFW	IGHV4-34	IGHJ3	IGHM				IGKV3-15	IGKJ1	IGKC
	clonotype966	CARELGSSEYFQHW	IGHV4-34	IGHJ1	IGHM				IGKV3-20	IGKJ4	IGKC
	clonotype311	CARERWFGESIYWYFDLW	IGHV4-34	IGHJ2	IGHM				IGKV3-20	IGKJ1	IGKC
	clonotype3557	CARGVSLELGHGGDAFDIW	IGHV4-34	IGHJ3	IGHM				IGKV3-20	IGKJ4	IGKC
	clonotype2322	CARETTKEVTTIAPW	IGHV4-34	IGHJ3	IGHM				IGKV3-20	IGKJ1	IGKC
	clonotype1878	CARGNSGSPDPIYAFDIW	IGHV4-34	IGHJ3	IGHM				IGKV3-20	IGKJ2	IGKC
	clonotype1091	CARVSQGTVVEAFDIW	IGHV4-34	IGHJ3	IGHM				IGKV3-20	IGKJ2	IGKC

	clonotype2361	CASERITMVRGSFDIW	IGHV4-34	IGHJ3	IGHM				IGKV3-20	IGKJ2	IGKC
	clonotype1661	CAKTYDSSGYYYGRAFDIW	IGHV4-34	IGHJ3	IGHM				IGKV3-20	IGKJ1	IGKC
	clonotype421	CARGSQEMATAFDYW	IGHV4-34	IGHJ4	IGHD				IGKV3-20	IGKJ2	IGKC
	clonotype1096	CARGLGVSGEMAAAGMGYW	IGHV4-34	IGHJ4	IGHM				IGKV3-20	IGKJ1	IGKC
	clonotype2805	CARKGIGGVDYW	IGHV4-34	IGHJ4	IGHM				IGKV3-20	IGKJ2	IGKC
	clonotype3452	CARGRGSSSFPHDYW	IGHV4-34	IGHJ4	IGHM				IGKV3-20	IGKJ3	IGKC
	clonotype1394	CARGRRAARPEVPLDYW	IGHV4-34	IGHJ4	IGHM				IGKV3-20	IGKJ1	IGKC
	clonotype740	CARLGRFVGATGVW	IGHV4-34	IGHJ4	IGHM				IGKV3-20	IGKJ1	IGKC
	clonotype524	CASSLAPLPGHYFDYW	IGHV4-34	IGHJ4	IGHM				IGKV3-20	IGKJ1	IGKC
	clonotype1865	CARGRRGYVVGPTVGPYFDSW	IGHV4-34	IGHJ4	IGHM				IGKV3-20	IGKJ2	IGKC
	clonotype2862	CARGGLSDYW	IGHV4-34	IGHJ4	IGHM				IGKV3-20	IGKJ1	IGKC
	clonotype2844	CARGQRRAVYYYDSSGLPFDYW	IGHV4-34	IGHJ4	IGHM				IGKV3-20	IGKJ2	IGKC
	clonotype121	CARRVELGYCSSTSCLNWFDPW	IGHV4-34	IGHJ5	IGHM				IGKV3-20	IGKJ2	IGKC
	clonotype274	CARGLNYYDSSGYIDPW	IGHV4-34	IGHJ5	IGHM				IGKV3-20	IGKJ1	IGKC
	clonotype1711	CARPQESKTGIAGRWFDPW	IGHV4-34	IGHJ5	IGHM				IGKV3-20	IGKJ2	IGKC
	clonotype1192	CAREGITGTTFFDPW	IGHV4-34	IGHJ5	IGHM				IGKV3-20	IGKJ1	IGKC
	clonotype71	CARGVQLWPLGMDVW	IGHV4-34	IGHJ6	IGHM				IGKV3-20	IGKJ2	IGKC
	clonotype3239	CARPPPDTVTTPLMDYGMDVW	IGHV4-34	IGHJ6	IGHD				IGKV3-20	IGKJ2	IGKC
	clonotype4044	CARVVSDYYYYGMDVW	IGHV4-34	IGHJ6	IGHD				IGKV3-20	IGKJ2	IGKC
	clonotype3570	CARNCSSTSCYPDSSSPGGVDVW	IGHV4-34	IGHJ6	IGHM				IGKV3-20	IGKJ2	IGKC
	clonotype3441	CARGLGYSSSGYYYYGMDVW	IGHV4-34	IGHJ6	IGHM				IGKV3-20	IGKJ2	IGKC
	clonotype1296	CARVVPAAPDRGWFDPW	IGHV4-34	IGHJ5	IGHM				IGKV3D-15	IGKJ4	IGKC
	clonotype1145	CARQEHIVVVTAIGGFDPW	IGHV4-34	IGHJ5	IGHM	IGLV2-14	IGLJ3	IGLC2	IGKV3D-15	IGKJ1	IGKC
	clonotype3672	CARGGMITFGGVIVALSYFDLW	IGHV4-34	IGHJ2	IGHM				IGKV4-1	IGKJ1	IGKC
	clonotype1476	CARRIAAAYAFDIW	IGHV4-34	IGHJ3	IGHM				IGKV4-1	IGKJ4	IGKC
	clonotype1611	CARGRYDFDYFDYW	IGHV4-34	IGHJ4	IGHM				IGKV4-1	IGKJ2	IGKC
	clonotype594	CASLSGPGFDYW	IGHV4-34	IGHJ4	IGHM				IGKV4-1	IGKJ1	IGKC
	clonotype3073	CARGSTVVTSTLNYW	IGHV4-34	IGHJ4	IGHM	IGLV4-69	IGLJ3	IGLC2	IGKV4-1	IGKJ3	IGKC
	clonotype2246	CARAAAAGTDGGWFDPW	IGHV4-34	IGHJ5	IGHD				IGKV4-1	IGKJ1	IGKC
	clonotype2184	CAKDRNGFDPW	IGHV4-34	IGHJ5	IGHM				IGKV4-1	IGKJ2	IGKC
	clonotype518	CASRYGDRPYYYYGMDVW	IGHV4-34	IGHJ6	IGHM				IGKV4-1	IGKJ3	IGKC
	clonotype802	CARGRVLFDYW	IGHV4-34	IGHJ4	IGHM				IGKV6-21	IGKJ1	IGKC
	clonotype155	CANLPFGQQLETLYFQHW	IGHV4-34	IGHJ1	IGHM	IGLV1-40	IGLJ3	IGLC2			
	clonotype2429	CARGGGQQLVVDAFDIW	IGHV4-34	IGHJ3	IGHM	IGLV1-40	IGLJ3	IGLC2			
	clonotype1569	CARKCSSKRWLPNPGYFDYW	IGHV4-34	IGHJ4	IGHM	IGLV1-40	IGLJ2	IGLC2			
	clonotype3923	CARIGTHYGDPTEYYFDYW	IGHV4-34	IGHJ4	IGHM	IGLV1-40	IGLJ3	IGLC2			
	clonotype671	CARGLGTAMATGGFDYW	IGHV4-34	IGHJ4	IGHM	IGLV1-40	IGLJ3	IGLC2			
	clonotype2525	CARGRPEFSTWYYFDYW	IGHV4-34	IGHJ4	IGHM	IGLV1-40	IGLJ1	IGLC1			
	clonotype2747	CARLGRFVGATGVW	IGHV4-34	IGHJ4	IGHM	IGLV1-40	IGLJ3	IGLC2			
	clonotype3479	CARGPVAAVYYFDYW	IGHV4-34	IGHJ4	IGHM	IGLV1-40	IGLJ1	IGLC1			
	clonotype1747	CARLTRSTYYYDSSGYYHFDYW	IGHV4-34	IGHJ4	IGHM	IGLV1-40	IGLJ1	IGLC1			
	clonotype3126	CARGVGSSWSGWFDPW	IGHV4-34	IGHJ5	IGHM	IGLV1-40	IGLJ3	IGLC2			
	clonotype3977	CARGDSSSSVKWFDPW	IGHV4-34	IGHJ5	IGHM	IGLV1-40	IGLJ3	IGLC2			
	clonotype2758	CARALDEYIVATTRGHWFDPW	IGHV4-34	IGHJ5	IGHM	IGLV1-40	IGLJ2	IGLC2			
	clonotype462	CARGRSDGADTPRGRFDPW	IGHV4-34	IGHJ5	IGHM	IGLV1-40	IGLJ2	IGLC2			
	clonotype2846	CARVRGSGITGTTRYFDYW	IGHV4-34	IGHJ4	IGHM	IGLV1-40;IGLV3-10	IGLJ2;IGLJ3	IGLC2;IGLC3			
	clonotype2632	CARASRGAVAGIRDW	IGHV4-34	IGHJ4	IGHM	IGLV1-40;IGLV3-21	IGLJ2;IGLJ3	IGLC2;IGLC2			
	clonotype2600	CARAPLGQLAGDWYFDRW	IGHV4-34	IGHJ2	IGHM	IGLV1-44	IGLJ1	IGLC1			
	clonotype1728	CASCERGGCSGYW	IGHV4-34	IGHJ4	IGHM	IGLV1-44	IGLJ2	IGLC2			
	clonotype1336	CVRNGTKGLDSW	IGHV4-34	IGHJ4	IGHM	IGLV1-44	IGLJ3	IGLC2			
	clonotype3899	CAPQGTGDPNYFDYW	IGHV4-34	IGHJ4	IGHM	IGLV1-44	IGLJ3	IGLC2			
	clonotype714	CARRTDDSSGYYFDYW	IGHV4-34	IGHJ4	IGHM	IGLV1-44	IGLJ7	IGLC7			
	clonotype1664	CASDPPGRVGMDVW	IGHV4-34	IGHJ6	IGHD	IGLV1-44	IGLJ1	IGLC1			
	clonotype363	CARDRQQLALGYYGMDVW	IGHV4-34	IGHJ6	IGHM	IGLV1-44	IGLJ1	IGLC1			

	clonotype1421	CARVVETTVTTQHRYGMDVW	IGHV4-34	IGHJ6	IGHM	IGLV1-44	IGLJ1	IGLC1			
	clonotype475	CARGIVVVPAARDGFDIW	IGHV4-34	IGHJ3	IGHM	IGLV1-47	IGLJ3	IGLC2			
	clonotype2263	CASLALRRPRITMVRGVDDAFDIW	IGHV4-34	IGHJ3	IGHM	IGLV1-47	IGLJ1	IGLC1			
	clonotype2547	CASLVTTDLPFDYW	IGHV4-34	IGHJ4	IGHM	IGLV1-47	IGLJ3	IGLC2			
	clonotype1112	CARGGGMVRGGLTFDYW	IGHV4-34	IGHJ4	IGHM	IGLV1-47;IGLV2-23	IGLJ2;IGLJ2	IGLC2;IGLC2			
	clonotype2395	CARRGEDSGDDCFDYW	IGHV4-34	IGHJ4	IGHM	IGLV1-47;IGLV9-49	IGLJ7;IGLJ3	IGLC7;IGLC2			
	clonotype1598	CARALGVAAGYWYFDLW	IGHV4-34	IGHJ2	IGHM	IGLV1-51	IGLJ2	IGLC2			
	clonotype3713	CAREMRQWLRWYFDLW	IGHV4-34	IGHJ2	IGHM	IGLV1-51	IGLJ3	IGLC3			
	clonotype3228	CARWTMNYFDYW	IGHV4-34	IGHJ4	IGHM	IGLV1-51	IGLJ2	IGLC2			
	clonotype1509	CAASIGGNSPRYW	IGHV4-34	IGHJ4	IGHM	IGLV1-51	IGLJ2	IGLC2			
	clonotype1391	CARGLKTVWGSPGYW	IGHV4-34	IGHJ4	IGHM	IGLV1-51	IGLJ3	IGLC3			
	clonotype2318	CARGYPLAATGTVGWFDPW	IGHV4-34	IGHJ5	IGHD	IGLV1-51	IGLJ3	IGLC2			
	clonotype3290	CARGRRGYSSGSNWFDPW	IGHV4-34	IGHJ5	IGHM	IGLV1-51	IGLJ1	IGLC1			
	clonotype244	CARLERGSITIFGGGYYYGMDVW	IGHV4-34	IGHJ6	IGHM	IGLV1-51	IGLJ3	IGLC2			
	clonotype3799	CARASGGGGEPGLGMDVW	IGHV4-34	IGHJ6	IGHD	IGLV1-51	IGLJ1	IGLC1			
	clonotype925	CARGRSSSSEGYYYYGMDVW	IGHV4-34	IGHJ6	IGHM	IGLV1-51	IGLJ2	IGLC2			
	clonotype3170	CAAYSSGQKIDYW	IGHV4-34	IGHJ4	IGHM	IGLV1-51;IGLV2-8	IGLJ1;IGLJ1	IGLC1;IGLC1			
	clonotype2244	CARPGEDW	IGHV4-34	IGHJ4	IGHM	IGLV10-54	IGLJ3	IGLC3			
	clonotype1357	CATLWNSSSWDKDYW	IGHV4-34	IGHJ4	IGHM	IGLV2-11	IGLJ1	IGLC1			
	clonotype809	CARGRRQYYDSSGYYLQHW	IGHV4-34	IGHJ1	IGHM	IGLV2-14	IGLJ2	IGLC2			
	clonotype1584	CARGAGRYSYRFWYFDLW	IGHV4-34	IGHJ2	IGHM	IGLV2-14	IGLJ3	IGLC3			
	clonotype2433	CARNTVGATAFDIW	IGHV4-34	IGHJ3	IGHM	IGLV2-14	IGLJ3	IGLC3			
	clonotype693	CARLVGGLRSSNPYYFDYW	IGHV4-34	IGHJ4	IGHM	IGLV2-14	IGLJ2	IGLC2			
	clonotype2566	CARGPTGRSNLGSDSSGYSLDYW	IGHV4-34	IGHJ4	IGHM	IGLV2-14	IGLJ3	IGLC3			
	clonotype1432	CARRGVDTAMATDFDYW	IGHV4-34	IGHJ4	IGHM	IGLV2-14	IGLJ1	IGLC1			
	clonotype2744	CARGGFITGTTYFDYW	IGHV4-34	IGHJ4	IGHM	IGLV2-14	IGLJ1	IGLC1			
	clonotype3589	CARGGTAAAGTNDYW	IGHV4-34	IGHJ4	IGHM	IGLV2-14	IGLJ2	IGLC2			
	clonotype3203	CARGVRFFDRLSLGHYFDYW	IGHV4-34	IGHJ4	IGHM	IGLV2-14	IGLJ2	IGLC2			
	clonotype2313	CARGWGYSSGWYRGYFDYW	IGHV4-34	IGHJ4	IGHM	IGLV2-14	IGLJ1	IGLC1			
	clonotype2843	CARTEVLLWFGEFPNWFDPW	IGHV4-34	IGHJ5	IGHM	IGLV2-14	IGLJ2	IGLC2			
	clonotype3722	CASWQLRDYYYGMDVW	IGHV4-34	IGHJ6	IGHD	IGLV2-14	IGLJ1	IGLC1			
	clonotype2197	CARGPTLPLTIFYGMDVW	IGHV4-34	IGHJ6	IGHM	IGLV2-14	IGLJ3	IGLC3			
	clonotype2452	CARGGAARLYGMDVW	IGHV4-34	IGHJ6	IGHM	IGLV2-14	IGLJ3	IGLC2			
	clonotype1312	CARGRPMNNYYYYAMDVW	IGHV4-34	IGHJ6	IGHM	IGLV2-14	IGLJ2	IGLC2			
	clonotype1470	CARGHPYCSGGSCDGDGMDVW	IGHV4-34	IGHJ6	IGHM	IGLV2-14	IGLJ3	IGLC3			
	clonotype4076	CARGRGRITMVRGVSDYGMDVW	IGHV4-34	IGHJ6	IGHM	IGLV2-14	IGLJ2	IGLC2			
	clonotype3004	CARGRDDSSGYYYAEVDVW	IGHV4-34	IGHJ6	IGHM	IGLV2-14	IGLJ3	IGLC3			
	clonotype2931	CARGPPTTVTTGFDPW	IGHV4-34	IGHJ5	IGHM	IGLV2-14;IGLV6-57	IGLJ3;IGLJ1	IGLC2;IGLC1			
	clonotype1212	CAREPRGVAVDYW	IGHV4-34	IGHJ4	IGHM	IGLV2-14;IGLV7-43	IGLJ3;IGLJ3	IGLC2;IGLC3			
	clonotype2013	CARRRESSSRYFDLW	IGHV4-34	IGHJ2	IGHM	IGLV2-23	IGLJ3	IGLC2			
	clonotype3748	CARKSRRLPPRWYFDLW	IGHV4-34	IGHJ2	IGHM	IGLV2-23	IGLJ3	IGLC2			
	clonotype1762	CARGSPYSSGWLKSRTWPYFDYW	IGHV4-34	IGHJ4	IGHD	IGLV2-23	IGLJ3	IGLC3			
	clonotype924	CASRGDSVKLYGGGRGWFDPW	IGHV4-34	IGHJ5	IGHM	IGLV2-23	IGLJ3	IGLC2			
	clonotype2054	CARGGGSSSWYGEHW	IGHV4-34	IGHJ1	IGHM	IGLV2-8	IGLJ2	IGLC2			
	clonotype2735	CARGSGVVVVVPVAAFDIW	IGHV4-34	IGHJ3	IGHM	IGLV2-8	IGLJ3	IGLC3			
	clonotype2218	CARGLGGLNDYVWGARAQGAFDIW	IGHV4-34	IGHJ3	IGHM	IGLV2-8	IGLJ2	IGLC2			
	clonotype1083	CARNGGRGYGYYYYGMDVW	IGHV4-34	IGHJ6	IGHM	IGLV2-8	IGLJ1	IGLC1			
	clonotype2495	CGRGSQVFRTRYYGMDVW	IGHV4-34	IGHJ6	IGHM	IGLV2-8	IGLJ2	IGLC2			
	clonotype1675	CARLANWGSGLDYW	IGHV4-34	IGHJ4	IGHM	IGLV2-8;IGLV6-57	IGLJ1;IGLJ3	IGLC1;IGLC2			
	clonotype641	CARGGGITGTTEYFDYW	IGHV4-34	IGHJ4	IGHM	IGLV3-1	IGLJ2	IGLC2			
	clonotype3801	CARLQRVRARSDRGYYYGSGSYDYW	IGHV4-34	IGHJ4	IGHM	IGLV3-10	IGLJ2	IGLC2			
	clonotype3687	CARGDNTYYYGSGAPDYW	IGHV4-34	IGHJ4	IGHM	IGLV3-10	IGLJ3	IGLC2			
	clonotype2954	CARGPVVVITNDAFDIW	IGHV4-34	IGHJ3	IGHM	IGLV3-19	IGLJ3	IGLC3			
	clonotype911	CARGYNTAAAGIDYW	IGHV4-34	IGHJ4	IGHM	IGLV3-19	IGLJ2	IGLC2			

	clonotype1841	CARGLLAVAPTGFGYW	IGHV4-34	IGHJ4	IGHM	IGLV3-19	IGLJ1	IGLC1			
	clonotype1758	CARVPSYSSSFFDYW	IGHV4-34	IGHJ4	IGHM	IGLV3-19	IGLJ2	IGLC2			
	clonotype2840	CARGYVLEEGTVIQLWLGYYFDYW	IGHV4-34	IGHJ4	IGHM	IGLV3-19	IGLJ1	IGLC1			
	clonotype1454	CARGRVAAAEYYFDYW	IGHV4-34	IGHJ4	IGHM	IGLV3-19	IGLJ2	IGLC2			
	clonotype1224	CARGSLLYYDFWSGSLFDYW	IGHV4-34	IGHJ4	IGHM	IGLV3-19	IGLJ2	IGLC2			
	clonotype4117	CARRFPAPGIAVAGTGDPFDYW	IGHV4-34	IGHJ4	IGHM	IGLV3-19	IGLJ3	IGLC3			
	clonotype3802	CARGYSGSYEGGDAFDIW	IGHV4-34	IGHJ3	IGHM	IGLV3-25	IGLJ2	IGLC2			
	clonotype1069	CARAQSRSGSYIRGFDYW	IGHV4-34	IGHJ4	IGHM	IGLV3-25	IGLJ2	IGLC2			
	clonotype976	CARVNPDYGGNSRGVDYW	IGHV4-34	IGHJ4	IGHM	IGLV3-9	IGLJ2	IGLC2			
	clonotype194	CARGRSRWLSNGGGDYW	IGHV4-34	IGHJ4	IGHM	IGLV7-43	IGLJ3	IGLC3			
	clonotype11	CASAGRPEYSYGWNW	IGHV4-34	IGHJ4	IGHM	IGLV7-43	IGLJ3	IGLC2			
	clonotype4111	CARGPNYYDSSGSLGYW	IGHV4-34	IGHJ4	IGHM	IGLV7-43	IGLJ3	IGLC3			
	clonotype885	CARGPSRWGDDILTGRPFDIW	IGHV4-34	IGHJ3	IGHM	IGLV7-46	IGLJ3	IGLC3			

**Sample**	**clonotype_id**	**igh_cdr3s**	**igh_v–genes**	**igh_j**	**igh_c**	**igl_v–genes**	**igl_j**	**igl_c**	**igk_v_genes**	**igk_j**	**igk_c**
**HC-07**	clonotype1744	CATPQGSSGPLGDAFDIW	IGHV4-34	IGHJ3	IGHM				IGKV1-5	IGKJ2	IGKC
	clonotype3110	CARGVPAAPFDYW	IGHV4-34	IGHJ4	IGHM				IGKV1-5	IGKJ2	IGKC
	clonotype3081	CARTGSSSWTSIVDYW	IGHV4-34	IGHJ4	IGHM				IGKV1-5	IGKJ2	IGKC
	clonotype2900	CARGSIAAAYYYYYMDVW	IGHV4-34	IGHJ6	IGHD				IGKV1-5	IGKJ4	IGKC
	clonotype3324	CARDRGSMTTVTTSWFDPW	IGHV4-34	IGHJ5	IGHM				IGKV1-6	IGKJ3	IGKC
	clonotype3269	CARGRGSSWVNAFDIW	IGHV4-34	IGHJ3	IGHM				IGKV1-8	IGKJ1	IGKC
	clonotype2804	CARGRYSGSYSLSGYFDYW	IGHV4-34	IGHJ4	IGHM				IGKV1-8	IGKJ1	IGKC
	clonotype672	CARGPLLWGCSSTSCYTGYFDYW	IGHV4-34	IGHJ4	IGHM				IGKV1-8	IGKJ1	IGKC
	clonotype2131	CARGYGSGSYYSFDYW	IGHV4-34	IGHJ4	IGHM				IGKV1-8	IGKJ1	IGKC
	clonotype2058	CASGFYGGNKRKLGDGYW	IGHV4-34	IGHJ4	IGHM				IGKV1-9	IGKJ5	IGKC
	clonotype3292	CAREGLELLPHYMDVW	IGHV4-34	IGHJ6	IGHM				IGKV1-9	IGKJ1	IGKC
	clonotype2297	CARGRICGYVCSYYYMDVW	IGHV4-34	IGHJ6	IGHM				IGKV1-12	IGKJ5	IGKC
	clonotype2266	CARGGGEQLVLPDYW	IGHV4-34	IGHJ4	IGHM				IGKV1-17	IGKJ2	IGKC
	clonotype2668	CARGLSVAARHRLGAFPYW	IGHV4-34	IGHJ4	IGHM				IGKV1-27	IGKJ1	IGKC
	clonotype2858	CARGGFWSGYYFW	IGHV4-34	IGHJ4	IGHM				IGKV1-27	IGKJ1	IGKC
	clonotype3016	CARVGVPAANW	IGHV4-34	IGHJ4	IGHM				IGKV1-33	IGKJ1	IGKC
	clonotype279	CARGHRYDFWSGYYGPHLGSAWFDPW	IGHV4-34	IGHJ5	IGHM				IGKV1-33	IGKJ2	IGKC
	clonotype2991	CARGSGFRELSLGYW	IGHV4-34	IGHJ4	IGHM				IGKV1D-8	IGKJ2	IGKC
	clonotype2682	CARARAFWSGYSPITGYYYYYMDVW	IGHV4-34	IGHJ6	IGHM				IGKV1D-8	IGKJ4	IGKC
	clonotype2960	CARGKYSSSLYYYYYMDVW	IGHV4-34	IGHJ6	IGHD				IGKV1D-13	IGKJ1	IGKC
	clonotype901	CARGRGEVVPAAPFDYW	IGHV4-34	IGHJ4	IGHM				IGKV1D-17	IGKJ3	IGKC
	clonotype1612	CARGNQLEIVVPGRRGAFDIW	IGHV4-34	IGHJ3	IGHM				IGKV1D-39	IGKJ2	IGKC
	clonotype2233	CARGGFDFWSGYVGYHDAFDIW	IGHV4-34	IGHJ3	IGHM				IGKV1D-39	IGKJ2	IGKC
	clonotype449	CARRRVWFREFLFDYW	IGHV4-34	IGHJ4	IGHM				IGKV1D-39	IGKJ3	IGKC
	clonotype2198	CARVPLRLSTRPYYYYGMDVW	IGHV4-34	IGHJ6	IGHM	IGLV1-47	IGLJ2	IGLC2	IGKV1D-39	IGKJ2	IGKC
	clonotype1221	CARGLSRWISSRGETRRNTRGWFDPW	IGHV4-34	IGHJ5	IGHD				IGKV1D-39	IGKJ4	IGKC
	clonotype293	CARLDYERGYALGYLDVW	IGHV4-34	IGHJ6	IGHD				IGKV1D-39	IGKJ1	IGKC
	clonotype3076	CAREAGRQLWLRGYGYW	IGHV4-34	IGHJ4	IGHM				IGKV2-28	IGKJ1	IGKC
	clonotype2469	CAVRAVIAAAGTRWFDPW	IGHV4-34	IGHJ5	IGHM				IGKV2-28	IGKJ1	IGKC
	clonotype605	CARGPRVENYYDSSGQYYYYYGMDVW	IGHV4-34	IGHJ6	IGHM				IGKV2-28	IGKJ2	IGKC
	clonotype2450	CAREENYITMVQGVSLRGWFDPW	IGHV4-34	IGHJ5	IGHD				IGKV2-28	IGKJ2	IGKC
	clonotype2673	CARGDRVLVVYAIRAGWYFDYW	IGHV4-34	IGHJ4	IGHM				IGKV2D-29;IGKV3-11	IGKJ3;IGKJ4	IGKC;IGKC
	clonotype614	CARGHGGPFGYW	IGHV4-34	IGHJ4	IGHM				IGKV3-11	IGKJ5	IGKC
	clonotype623	CARGGQQLVPQRFDYW	IGHV4-34	IGHJ4	IGHD				IGKV3-11	IGKJ3	IGKC
	clonotype1811	CARERLSTAGHDYFYYYMDVW	IGHV4-34	IGHJ6	IGHM				IGKV3-11;IGKV3-15	IGKJ5;IGKJ1	IGKC;IGKC
	clonotype488	CARGLTAPSRPSSYSSSWYRSYW	IGHV4-34	IGHJ4	IGHM				IGKV3-15	IGKJ1	IGKC
	clonotype881	CARGKNYYYYYMDVW	IGHV4-34	IGHJ6	IGHM				IGKV3-15	IGKJ4	IGKC
	clonotype3140	CAVRLVTSNIRYYYYYGMDVW	IGHV4-34	IGHJ6	IGHM				IGKV3-15	IGKJ1	IGKC

	clonotype2089	CARGLRT	IGHV4-34	IGHJ6	IGHM				IGKV3-15	IGKJ2	IGKC
	clonotype1043	CARVLRYLRWFDYW	IGHV4-34	IGHJ4	IGHM				IGKV3-20	IGKJ2	IGKC
	clonotype143	CARGPSKISYYYYYMDVW	IGHV4-34	IGHJ6	IGHM				IGKV3-20	IGKJ2	IGKC
	clonotype2313	CARGTFSGYDYWDYW	IGHV4-34	IGHJ4	IGHM				IGKV4-1	IGKJ4	IGKC
	clonotype1416	CARGTYSGYDYWDYW	IGHV4-34	IGHJ4	IGHM				IGKV4-1	IGKJ4	IGKC
	clonotype657	CARGLVSGTNMGPFDYW	IGHV4-34	IGHJ4	IGHM				IGKV4-1	IGKJ2	IGKC
	clonotype2921	CARGRKAIPKYNWFDPW	IGHV4-34	IGHJ5	IGHM				IGKV4-1	IGKJ1	IGKC
	clonotype3182	CARVLTGLMPYYMDVW	IGHV4-34	IGHJ6	IGHM				IGKV4-1	IGKJ2	IGKC
	clonotype1116	CARVGAGTDYW	IGHV4-34	IGHJ4	IGHD				IGKV4-1	IGKJ4	IGKC
	clonotype2962	CARVGSWYGSGSYPFDYW	IGHV4-34	IGHJ4	IGHM	IGLV1-40	IGLJ2	IGLC2			
	clonotype936	CARHPYYDFWSGYSPIHPRYNWFDPW	IGHV4-34	IGHJ5	IGHM	IGLV1-40	IGLJ2	IGLC2			
	clonotype2294	CARGLGATSAFDYW	IGHV4-34	IGHJ4	IGHM	IGLV1-44	IGLJ2	IGLC2			
	clonotype1836	CARVIFCCAPRYADSYFQHW	IGHV4-34	IGHJ1	IGHM	IGLV1-47	IGLJ3	IGLC3			
	clonotype3127	CARDQPPKLFDYW	IGHV4-34	IGHJ4	IGHM	IGLV1-47	IGLJ1	IGLC1			
	clonotype768	CARRGVLGYCSGGSCTYYYGMDVW	IGHV4-34	IGHJ6	IGHM	IGLV1-47	IGLJ2	IGLC2			
	clonotype2909	CASRAYYYGSGSYFPKTGFYYYYGMDVW	IGHV4-34	IGHJ6	IGHM	IGLV1-47	IGLJ2	IGLC2			
	clonotype1535	CARFLGGVISLDYW	IGHV4-34	IGHJ4	IGHM	IGLV1-47;IGLV2-23	IGLJ2;IGLJ2	IGLC2;IGLC2			
	clonotype1919	CARRGPRGRPWLHRNWFDPW	IGHV4-34	IGHJ5	IGHM	IGLV1-51	IGLJ3	IGLC3			
	clonotype1030	CARVVRDLMRTYYYYMDVW	IGHV4-34	IGHJ6	IGHM	IGLV1-51	IGLJ2	IGLC2			
	clonotype1142	CARGPYSSSTPWRAYFDYW	IGHV4-34	IGHJ4	IGHM	IGLV1-51;IGLV6-57	IGLJ2;IGLJ2	IGLC2;IGLC2			
	clonotype2712	CARAQLRFKFDYW	IGHV4-34	IGHJ4	IGHM	IGLV2-11	IGLJ1	IGLC1			
	clonotype3315	CARTPFLGGWFDPW	IGHV4-34	IGHJ5	IGHM	IGLV2-11	IGLJ1	IGLC1			
	clonotype933	CARALTTFSSSGHRLDWHFDLW	IGHV4-34	IGHJ2	IGHM	IGLV2-14	IGLJ2	IGLC2			
	clonotype1204	CARGRFWSGFLW	IGHV4-34	IGHJ4	IGHM	IGLV2-14	IGLJ2	IGLC2			
	clonotype25	CARGSIVVVPAAIIGVSAFDPW	IGHV4-34	IGHJ5	IGHM	IGLV2-14	IGLJ3	IGLC3			
	clonotype2461	CARGRITIFGVASHNWFDPW	IGHV4-34	IGHJ5	IGHM	IGLV2-14	IGLJ2	IGLC2			
	clonotype2742	CARADPYYPEGVRGVRFDPW	IGHV4-34	IGHJ5	IGHM	IGLV3-1	IGLJ2	IGLC2			
	clonotype74	CARGPLCSSTSCYPSYYGMDVW	IGHV4-34	IGHJ6	IGHD	IGLV3-1	IGLJ2	IGLC2			
	clonotype2397	CARAHTLTTVTTRELDYW	IGHV4-34	IGHJ4	IGHM	IGLV3-19	IGLJ2	IGLC2			
	clonotype1034	CARGPGYSGYDFYPNW	IGHV4-34	IGHJ4	IGHM	IGLV3-19	IGLJ2	IGLC3			
	clonotype1911	CARGLYSGSYWGVKGRFDPW	IGHV4-34	IGHJ5	IGHM	IGLV3-19	IGLJ2	IGLC2			
	clonotype1496	CARVSVVVPALSDPW	IGHV4-34	IGHJ5	IGHM	IGLV3-19	IGLJ2	IGLC2			
	clonotype1865	CARGLGNDYGDYDYYYYGMDVW	IGHV4-34	IGHJ6	IGHD	IGLV3-25	IGLJ3	IGLC3			
**Sample**	**clonotype_id**	**igh_cdr3s**	**igh_v–genes**	**igh_j**	**igh_c**	**igl_v–genes**	**igl_j**	**igl_c**	**igk_v_genes**	**igk_j**	**igk_c**
**HC-15**	clonotype2434	CARERELLRGDAFDIW	IGHV4-34	IGHJ3	IGHM				IGKV1-5	IGKJ1	IGKC
	clonotype3110	CARGHVRKRGGSGSYYKFPFDYW	IGHV4-34	IGHJ4	IGHM				IGKV1-5	IGKJ1	IGKC
	clonotype297	CARGFWSKGKRAAILFDYW	IGHV4-34	IGHJ4	IGHM				IGKV1-5	IGKJ4	IGKC
	clonotype2251	CARGGYSGSGLYAPLRGW	IGHV4-34	IGHJ4	IGHM				IGKV1-5	IGKJ1	IGKC
	clonotype285	CAGGGGKILPFDYW	IGHV4-34	IGHJ4	IGHM				IGKV1-5	IGKJ1	IGKC
	clonotype3198	CARGLGRVVGSYFMFLGAYYFDYW	IGHV4-34	IGHJ4	IGHM				IGKV1-5	IGKJ1	IGKC
	clonotype947	CARGYFWLMAVAGYYFDYW	IGHV4-34	IGHJ4	IGHM				IGKV1-5	IGKJ1	IGKC
	clonotype320	CARLRPYGSGSYNLKYYFDYW	IGHV4-34	IGHJ4	IGHM				IGKV1-5	IGKJ1	IGKC
	clonotype5348	CARGLLITFGGVIAKPIDYW	IGHV4-34	IGHJ4	IGHM				IGKV1-5	IGKJ2	IGKC
	clonotype3346	CARGPLRREGPPRGYSKPW	IGHV4-34	IGHJ5	IGHM				IGKV1-5	IGKJ2	IGKC
	clonotype4683	CARQNDSEYYYYYGMDVW	IGHV4-34	IGHJ6	IGHM				IGKV1-5	IGKJ2	IGKC
	clonotype4444	CAREHRSLYCSSTSCYRPYYYYGMDVW	IGHV4-34	IGHJ6	IGHM				IGKV1-5	IGKJ4	IGKC
	clonotype299	CARRYSLYYFDYW	IGHV4-34	IGHJ4	IGHM				IGKV1-5;IGKV3-11	IGKJ1;IGKJ5	IGKC;IGKC
	clonotype32	CARGRLVTARVEYFQHW	IGHV4-34	IGHJ1	IGHM				IGKV1-6	IGKJ1	IGKC
	clonotype3652	CARKAGHRRITVAANYYYYGMDVW	IGHV4-34	IGHJ6	IGHM				IGKV1-6	IGKJ2	IGKC
	clonotype4088	CASGLRNWNDVSSRFDPW	IGHV4-34	IGHJ5	IGHM				IGKV1-8	IGKJ4	IGKC
	clonotype1632	CARGDYYGSGSLIWGSGWFDPW	IGHV4-34	IGHJ5	IGHM				IGKV1-8	IGKJ1	IGKC
	clonotype65	CASLPRYSYGYYGVPNYYGMDVW	IGHV4-34	IGHJ6	IGHM				IGKV1-8	IGKJ4	IGKC
	clonotype64	CARDPRKDGYCSSTSCPKYYYYYGMDVW	IGHV4-34	IGHJ6	IGHM				IGKV1-8	IGKJ1	IGKC

	clonotype2147	CARFRYCSSTSCYTRWDYYYGMDVW	IGHV4-34	IGHJ6	IGHM				IGKV1-8	IGKJ4	IGKC
	clonotype2114	CARTPNIIARPFDYW	IGHV4-34	IGHJ4	IGHM				IGKV1-12	IGKJ1	IGKC
	clonotype1986	CARGTVTEDYW	IGHV4-34	IGHJ4	IGHM				IGKV1-16	IGKJ1	IGKC
	clonotype243	CARGYYTVTSAYW	IGHV4-34	IGHJ4	IGHM				IGKV1-16	IGKJ2	IGKC
	clonotype4584	CARLPWLVRYFDYW	IGHV4-34	IGHJ4	IGHM				IGKV1-17	IGKJ1	IGKC
	clonotype122	CARSPTGLRDYW	IGHV4-34	IGHJ4	IGHM				IGKV1-17	IGKJ2	IGKC
	clonotype4656	CARGLLGSKDYW	IGHV4-34	IGHJ4	IGHM				IGKV1-17	IGKJ1	IGKC
	clonotype622	CARGSSWLPVNW	IGHV4-34	IGHJ4	IGHM				IGKV1-17	IGKJ4	IGKC
	clonotype3514	CARGRSGYGSGSYDYW	IGHV4-34	IGHJ4	IGHM				IGKV1-17	IGKJ1	IGKC
	clonotype5128	CARVSKWVAYVGFDPW	IGHV4-34	IGHJ5	IGHM				IGKV1-17	IGKJ1	IGKC
	clonotype797	CARGGLSGTIIAARPGGMDVW	IGHV4-34	IGHJ6	IGHM				IGKV1-17	IGKJ2	IGKC
	clonotype2938	CARMSRIQLWLEANYYFYYGMDVW	IGHV4-34	IGHJ6	IGHM				IGKV1-17	IGKJ1	IGKC
	clonotype3302	CARVGGSGSYQGFHYYYYGMDVW	IGHV4-34	IGHJ6	IGHM				IGKV1-17	IGKJ3	IGKC
	clonotype3357	CARGPSRPRGVVVVPAAYNAFDIW	IGHV4-34	IGHJ3	IGHM				IGKV1-27	IGKJ4	IGKC
	clonotype5205	CARAAGYDSSQW	IGHV4-34	IGHJ4	IGHM				IGKV1-27	IGKJ1	IGKC
	clonotype865	CARGGRGLLRSPVYW	IGHV4-34	IGHJ4	IGHM				IGKV1-27	IGKJ4	IGKC
	clonotype2897	CASRRSGYSGYW	IGHV4-34	IGHJ4	IGHM				IGKV1-27	IGKJ1	IGKC
	clonotype4040	CAWGFYDILTGPLDAFDIW	IGHV4-34	IGHJ3	IGHM				IGKV1D-39	IGKJ1	IGKC
	clonotype473	CARSSAGGTTVFW	IGHV4-34	IGHJ4	IGHM				IGKV1D-39	IGKJ1	IGKC
	clonotype1398	CARVRLLRKTAMVIYYFDYW	IGHV4-34	IGHJ4	IGHM				IGKV1D-39	IGKJ2	IGKC
	clonotype5033	CARGVARRYYDRLHDYW	IGHV4-34	IGHJ4	IGHM				IGKV1D-39	IGKJ1	IGKC
	clonotype3753	CARRPRRDGYKRFRDYFDYW	IGHV4-34	IGHJ4	IGHM				IGKV1D-39	IGKJ2	IGKC
	clonotype1894	CARVPRFVYSSSSLYYFDYW	IGHV4-34	IGHJ4	IGHM				IGKV1D-39	IGKJ2	IGKC
	clonotype1572	CARGRQYCSSTSCYTGIIDRTYYFDYW	IGHV4-34	IGHJ4	IGHM				IGKV1D-39	IGKJ1	IGKC
	clonotype5374	CARPLYYYDSSGYSGW	IGHV4-34	IGHJ4	IGHM				IGKV1D-39	IGKJ2	IGKC
	clonotype864	CARDNYDFWSGYYTSGYDYW	IGHV4-34	IGHJ4	IGHM				IGKV1D-39	IGKJ4	IGKC
	clonotype3445	CARGRAGNGSGKRFDPW	IGHV4-34	IGHJ5	IGHM				IGKV1D-39	IGKJ2	IGKC
	clonotype2430	CARIRCSGGSCYPSWFDPW	IGHV4-34	IGHJ5	IGHM				IGKV1D-39	IGKJ2	IGKC
	clonotype2544	CARVQTYKQVMRTKNWFDPW	IGHV4-34	IGHJ5	IGHM				IGKV1D-39	IGKJ1	IGKC
	clonotype446	CARGRVAPVTFDPW	IGHV4-34	IGHJ5	IGHM				IGKV1D-39	IGKJ5	IGKC
	clonotype2321	CARGQDLRIFGAYYYYYYGMDVW	IGHV4-34	IGHJ6	IGHM				IGKV1D-39	IGKJ4	IGKC
	clonotype5048	CARVGFLEWLSDDHYYYGMDVW	IGHV4-34	IGHJ6	IGHM				IGKV1D-39	IGKJ2	IGKC
	clonotype4446	CARGQYDFWSGYYPDYYYYYGMDVW	IGHV4-34	IGHJ6	IGHM				IGKV1D-39	IGKJ3	IGKC
	clonotype3639	CARGDDFWSGYYLYWYFDLW	IGHV4-34	IGHJ2	IGHM				IGKV1D-39;IGKV4-1	IGKJ4;IGKJ2	IGKC;IGKC
	clonotype1288	CARGRRTARTHFDYW	IGHV4-34	IGHJ4	IGHM	IGLV2-14	IGLJ1	IGLC1	IGKV1D-43	IGKJ4	IGKC
	clonotype1038	CAFRTQKWNGFDYW	IGHV4-34	IGHJ4	IGHM				IGKV1D-8	IGKJ1	IGKC
	clonotype1221	CARGLPGYGSGSLTFSYW	IGHV4-34	IGHJ4	IGHM				IGKV2-24	IGKJ5	IGKC
	clonotype2806	CARSQRWLQVGHGFQHW	IGHV4-34	IGHJ1	IGHM				IGKV2-28	IGKJ5	IGKC
	clonotype3386	CARGLYCSSTSCYPYGFDPW	IGHV4-34	IGHJ5	IGHM				IGKV2-28	IGKJ2	IGKC
	clonotype337	CARVRVAAAYSRPYYYYGMDVW	IGHV4-34	IGHJ6	IGHM				IGKV2-28	IGKJ2	IGKC
	clonotype5366	CARGSGYDFWSGYTNYYYGMDVW	IGHV4-34	IGHJ6	IGHM				IGKV2-30	IGKJ1	IGKC
	clonotype5338	CARLKGRYSPAFVYW	IGHV4-34	IGHJ4	IGHM				IGKV2-30;IGKV4-1	IGKJ4;IGKJ4	IGKC;IGKC
	clonotype3160	CAGPNWNYASDDAFDIW	IGHV4-34	IGHJ3	IGHM				IGKV3-11	IGKJ2	IGKC
	clonotype3050	CARGLWLRLGYFDYW	IGHV4-34	IGHJ4	IGHM				IGKV3-11	IGKJ4	IGKC
	clonotype3591	CARGDSTYGPYYYGSGSYGTYW	IGHV4-34	IGHJ4	IGHM				IGKV3-11	IGKJ2	IGKC
	clonotype2323	CARRALLQRGIVVVTKNWFDPW	IGHV4-34	IGHJ5	IGHM				IGKV3-11	IGKJ2	IGKC
	clonotype3778	CARVLFRRSYYGSGSYRPGANWFDPW	IGHV4-34	IGHJ5	IGHM				IGKV3-11	IGKJ1	IGKC
	clonotype4256	CARGKRAYYDFWS	IGHV4-34	IGHJ1	IGHM				IGKV3-15	IGKJ2	IGKC
	clonotype4682	CASVWFLAFDIW	IGHV4-34	IGHJ3	IGHM				IGKV3-15	IGKJ5	IGKC
	clonotype3489	CARPRRKHPLYDSSGFFDYW	IGHV4-34	IGHJ4	IGHM				IGKV3-15	IGKJ2	IGKC
	clonotype2846	CARRLGYSSGWTSRYFDYW	IGHV4-34	IGHJ4	IGHM				IGKV3-15	IGKJ2	IGKC
	clonotype2582	CARSPPRYCSSSSCYKALDYW	IGHV4-34	IGHJ4	IGHM				IGKV3-15	IGKJ2	IGKC
	clonotype4193	CAKRKYGSGSYWLWWPFDYW	IGHV4-34	IGHJ4	IGHM				IGKV3-15	IGKJ2	IGKC
	clonotype2563	CARSKITMVRGVIITSYFDYW	IGHV4-34	IGHJ4	IGHM				IGKV3-15	IGKJ4	IGKC

	clonotype789	CARAVRDFWSGYQTTNDYW	IGHV4-34	IGHJ4	IGHM				IGKV3-15	IGKJ1	IGKC
	clonotype2096	CARGGRPFVSGVPTATRYWYFDIW	IGHV4-34	IGHJ2	IGHM				IGKV3-20	IGKJ5	IGKC
	clonotype2789	CARGLRLFLAAAGIW	IGHV4-34	IGHJ4	IGHM				IGKV3-20	IGKJ2	IGKC
	clonotype238	CARGPEQLYAYW	IGHV4-34	IGHJ4	IGHM				IGKV3-20	IGKJ1	IGKC
	clonotype5163	CARRGGVRRWLQQTPVDYW	IGHV4-34	IGHJ4	IGHM				IGKV3-20	IGKJ1	IGKC
	clonotype3603	CARGRIPDSGSYKDW	IGHV4-34	IGHJ4	IGHM				IGKV3-20	IGKJ1	IGKC
	clonotype3808	CARKNGGYYYVKPFDYW	IGHV4-34	IGHJ4	IGHM				IGKV3-20	IGKJ5	IGKC
	clonotype3168	CARGLAVVRGVFGYW	IGHV4-34	IGHJ4	IGHM				IGKV3-20	IGKJ3	IGKC
	clonotype1678	CARAYGSGSYLFYFDYW	IGHV4-34	IGHJ4	IGHM				IGKV3-20	IGKJ1	IGKC
	clonotype3091	CARGPVFGVVMIRPFQPW	IGHV4-34	IGHJ4	IGHM				IGKV3-20	IGKJ5	IGKC
	clonotype4405	CARGPIVVVPAAILVSGGVSYYFDYW	IGHV4-34	IGHJ4	IGHM				IGKV3-20	IGKJ4	IGKC
	clonotype4219	CARVRPYYYGSGFLKGPFDYW	IGHV4-34	IGHJ4	IGHM				IGKV3-20	IGKJ3	IGKC
	clonotype440	CARGGLWFGLRGKANWFDPW	IGHV4-34	IGHJ5	IGHM				IGKV3-20	IGKJ4	IGKC
	clonotype4062	CARGVSPWLFYWFDPW	IGHV4-34	IGHJ5	IGHM				IGKV3-20	IGKJ1	IGKC
	clonotype4764	CARVSRTTVTTGGWFDPW	IGHV4-34	IGHJ5	IGHM				IGKV3-20	IGKJ1	IGKC
	clonotype1862	CARGLRRVSGWYDYW	IGHV4-34	IGHJ5	IGHM				IGKV3-20	IGKJ5	IGKC
	clonotype3411	CARGGPQAGTVPVGGDYYYYGMDVW	IGHV4-34	IGHJ6	IGHM				IGKV3-20	IGKJ4	IGKC
	clonotype2263	CARGYPIVVVPAAIDRSLSYYYGMDVW	IGHV4-34	IGHJ6	IGHM				IGKV3-20	IGKJ1	IGKC
	clonotype4669	CARGNLFIRGYTHGGGDYW	IGHV4-34	IGHJ4	IGHM				IGKV3D-15	IGKJ1	IGKC
	clonotype4014	CAREVRLQLWLRGGMDVW	IGHV4-34	IGHJ6	IGHM				IGKV3D-15	IGKJ2	IGKC
	clonotype5039	CARRSGNSSGWYYPRPRAFDIW	IGHV4-34	IGHJ3	IGHM				IGKV4-1	IGKJ2	IGKC
	clonotype1683	CARGPLTPGAFDIW	IGHV4-34	IGHJ3	IGHM				IGKV4-1	IGKJ1	IGKC
	clonotype1310	CARVGPRGGYYKRAEFDYW	IGHV4-34	IGHJ4	IGHM				IGKV4-1	IGKJ4	IGKC
	clonotype2954	CARGRSRVYFDYW	IGHV4-34	IGHJ4	IGHM				IGKV4-1	IGKJ5	IGKC
	clonotype2068	CASTHFGVVTDYW	IGHV4-34	IGHJ4	IGHM				IGKV4-1	IGKJ4	IGKC
	clonotype604	CARGHRYNWFDPW	IGHV4-34	IGHJ5	IGHM				IGKV4-1	IGKJ4	IGKC
	clonotype2374	CARGRRQQRMGAWFDPW	IGHV4-34	IGHJ5	IGHM				IGKV4-1	IGKJ2	IGKC
	clonotype3229	CARGQPHRSWYAPGFDPW	IGHV4-34	IGHJ5	IGHM				IGKV4-1	IGKJ1	IGKC
	clonotype3218	CARDKGLGYWARNYYYGMDVW	IGHV4-34	IGHJ6	IGHM				IGKV4-1	IGKJ4	IGKC
	clonotype3247	CARSRSYYYGMDVW	IGHV4-34	IGHJ6	IGHM				IGKV4-1	IGKJ1	IGKC
	clonotype5145	CARGRTRYCSSTSCYRAYYYYYGMDVW	IGHV4-34	IGHJ6	IGHM				IGKV4-1	IGKJ1	IGKC
	clonotype4706	CARDCCLKDGYYVEDYW	IGHV4-34	IGHJ4	IGHM	IGLV1-40	IGLJ2	IGLC2			
	clonotype2790	CARGAALGYCSGGSCRHRRHRGWFDPW	IGHV4-34	IGHJ5	IGHM	IGLV1-40	IGLJ3	IGLC3			
	clonotype5332	CARGGSLPANPMDVW	IGHV4-34	IGHJ6	IGHM	IGLV1-40	IGLJ2	IGLC2			
	clonotype2350	CASLRYRAARYYYYGMDVW	IGHV4-34	IGHJ6	IGHM	IGLV1-40	IGLJ1	IGLC1			
	clonotype3983	CARGMVRGVITTPGGMDVW	IGHV4-34	IGHJ6	IGHM	IGLV1-40	IGLJ2	IGLC3			
	clonotype2511	CATLLGYCSGGSCYHNWFDPW	IGHV4-34	IGHJ5	IGHM	IGLV1-40;IGLV9-49	IGLJ1;IGLJ2	IGLC1;IGLC2			
	clonotype4914	CARSSIVVVVAAKNAFDIW	IGHV4-34	IGHJ3	IGHM	IGLV1-44	IGLJ2	IGLC2			
	clonotype1843	CAREGYSNYWFDPW	IGHV4-34	IGHJ5	IGHM	IGLV1-44	IGLJ3	IGLC3			
	clonotype3232	CARGLKWLVRGGYYGMDVW	IGHV4-34	IGHJ6	IGHM	IGLV1-44	IGLJ2	IGLC2			
	clonotype636	CARGVTIFGEGVPFGYW	IGHV4-34	IGHJ4	IGHM	IGLV1-47	IGLJ3	IGLC3			
	clonotype4421	CARVSSSWHKDYGMDVW	IGHV4-34	IGHJ6	IGHM	IGLV1-47	IGLJ2	IGLC2			
	clonotype2042	CARRPYYDFWSGYYTGLLPLGGMDVW	IGHV4-34	IGHJ6	IGHM	IGLV1-47	IGLJ2	IGLC3			
	clonotype42	CARGHRTRGYSYGYRNDAFDIW	IGHV4-34	IGHJ3	IGHM	IGLV1-51	IGLJ2	IGLC2			
	clonotype4477	CATSSIAARWLGPSSVGRAAPFDYW	IGHV4-34	IGHJ4	IGHM	IGLV1-51	IGLJ2	IGLC2			
	clonotype3331	CARYAPRAAQQWLVRVGYYFDYW	IGHV4-34	IGHJ4	IGHM	IGLV1-51	IGLJ1	IGLC1			
	clonotype3216	CASLYGSSAYFYYW	IGHV4-34	IGHJ4	IGHM	IGLV1-51	IGLJ2	IGLC3			
	clonotype1508	CARRVVVVAGTNNWFDPW	IGHV4-34	IGHJ5	IGHM	IGLV1-51	IGLJ2	IGLC3			
	clonotype4358	CARGGRTMTRRRNWFDPW	IGHV4-34	IGHJ5	IGHM	IGLV1-51	IGLJ3	IGLC3			
	clonotype1469	CARGRYGDKNWFDPW	IGHV4-34	IGHJ5	IGHM	IGLV1-51	IGLJ3	IGLC3			
	clonotype4462	CARGRGVWATRGYYYGLDVW	IGHV4-34	IGHJ6	IGHM	IGLV1-51	IGLJ1	IGLC1			
	clonotype593	CARSPRHFPSYSRPNAFDIW	IGHV4-34	IGHJ3	IGHM	IGLV2-11	IGLJ3	IGLC3			
	clonotype5289	CARWHRSHYYDSSGYYSRDYYFDYW	IGHV4-34	IGHJ4	IGHM	IGLV2-11	IGLJ2	IGLC2			
	clonotype4115	CAREHYDFWSGYYLVLDYW	IGHV4-34	IGHJ4	IGHM	IGLV2-11	IGLJ1	IGLC1			

	clonotype857	CARGHDFWSGYWGNWFDPW	IGHV4-34	IGHJ5	IGHM	IGLV2-11	IGLJ1	IGLC1			
	clonotype5130	CARNVVGGVAFEIW	IGHV4-34	IGHJ3	IGHM	IGLV2-14	IGLJ2	IGLC3			
	clonotype1614	CARGRYW	IGHV4-34	IGHJ4	IGHM	IGLV2-14	IGLJ2	IGLC2			
	clonotype1728	CARGPVVPAAIHYFDYW	IGHV4-34	IGHJ4	IGHM	IGLV2-14	IGLJ2	IGLC3			
	clonotype3721	CARVAGYYYDSSGYSYYFDYW	IGHV4-34	IGHJ4	IGHM	IGLV2-14	IGLJ1	IGLC1			
	clonotype1690	CARGPLNIGNWNYLPRPLYYYGMDVW	IGHV4-34	IGHJ6	IGHM	IGLV2-14	IGLJ3	IGLC3			
	clonotype2792	CARGSRDHSSGWYRFHYGMDVW	IGHV4-34	IGHJ6	IGHM	IGLV2-14	IGLJ1	IGLC1			
	clonotype4122	CQTRSWRGDPLKVW	IGHV4-34	IGHJ2	IGHM	IGLV2-23	IGLJ2	IGLC3			
	clonotype4798	CARGTAAYYFDYW	IGHV4-34	IGHJ4	IGHM	IGLV2-23	IGLJ1	IGLC1			
	clonotype792	CAGRIAVSEAGAYW	IGHV4-34	IGHJ4	IGHM	IGLV2-23	IGLJ2	IGLC3			
	clonotype784	CARSRGWYSGVRFDPW	IGHV4-34	IGHJ5	IGHM	IGLV2-23	IGLJ2	IGLC2			
	clonotype3989	CARGLVRFRRGGGYW	IGHV4-34	IGHJ4	IGHM	IGLV2-8	IGLJ2	IGLC2			
	clonotype940	CAAGLHARGDDAFDIW	IGHV4-34	IGHJ3	IGHM	IGLV3-1	IGLJ2	IGLC2			
	clonotype539	CARGRGDGYNLDYW	IGHV4-34	IGHJ4	IGHM	IGLV3-1	IGLJ2	IGLC2			
	clonotype920	CARGPWDIVVVPAAPPGWWFDPW	IGHV4-34	IGHJ5	IGHM	IGLV3-1	IGLJ2	IGLC2			
	clonotype4745	CAREPIPSLRVVNFSGFDVW	IGHV4-34	IGHJ6	IGHM	IGLV3-1	IGLJ2	IGLC2			
	clonotype1939	CALPPHRRYYDFWSGYSFQHW	IGHV4-34	IGHJ1	IGHM	IGLV3-19	IGLJ2	IGLC3			
	clonotype2551	CARGNTYDSRYFDLW	IGHV4-34	IGHJ2	IGHM	IGLV3-19	IGLJ2	IGLC2			
	clonotype318	CARAGIGVARPYYFDYW	IGHV4-34	IGHJ4	IGHM	IGLV3-19	IGLJ2	IGLC2			
	clonotype1577	CARPGRGWQQYTFDYW	IGHV4-34	IGHJ4	IGHM	IGLV3-19	IGLJ2	IGLC3			
	clonotype1479	CARGVIGYCSSTSCGLRIQLWLYFDYW	IGHV4-34	IGHJ4	IGHM	IGLV3-19	IGLJ3	IGLC3			
	clonotype383	CARGFYDSSGYYLSLFDYW	IGHV4-34	IGHJ4	IGHM	IGLV3-19	IGLJ1	IGLC1			
	clonotype3549	CARGGRGYDSSGYYGYW	IGHV4-34	IGHJ4	IGHM	IGLV3-19	IGLJ1	IGLC1			
	clonotype4907	CARGRKGVYILTGYLDYW	IGHV4-34	IGHJ4	IGHM	IGLV3-19	IGLJ2	IGLC2			
	clonotype1318	CARLWFTRKILYSYGYNRWFDPW	IGHV4-34	IGHJ5	IGHM	IGLV3-19	IGLJ2	IGLC2			
	clonotype2235	CARAVYYASWFDPW	IGHV4-34	IGHJ5	IGHM	IGLV3-19	IGLJ2	IGLC3			
	clonotype4325	CARAMGIVVVPAAINWFDPW	IGHV4-34	IGHJ5	IGHM	IGLV3-19	IGLJ2	IGLC3			
	clonotype1785	CARGGYSYGYDWFDPW	IGHV4-34	IGHJ5	IGHM	IGLV3-19	IGLJ2	IGLC2			
	clonotype1457	CARGRLWFGELHYYYYGMDVW	IGHV4-34	IGHJ6	IGHM	IGLV3-19	IGLJ2	IGLC2			
	clonotype4234	CARPRQGVW	IGHV4-34	IGHJ6	IGHM	IGLV3-19	IGLJ2	IGLC3			
	clonotype1578	CARDCYYGFYGMDVW	IGHV4-34	IGHJ6	IGHM	IGLV3-25	IGLJ3	IGLC3			
	clonotype715	CARAPRHYYYDSSGYYQGSREYYGMDVW	IGHV4-34	IGHJ6	IGHM	IGLV3-25	IGLJ2	IGLC3			
	clonotype4956	CARRLAVAGTVDYW	IGHV4-34	IGHJ4	IGHM	IGLV7-43	IGLJ3	IGLC3			
	clonotype2914	CARGLGWCSSTSCQPKKAMDVW	IGHV4-34	IGHJ6	IGHM	IGLV7-43	IGLJ3	IGLC3			
	clonotype1117	CARGHYDYVWGSYRNEPSPLRAVW	IGHV4-34	IGHJ6	IGHM	IGLV9-49	IGLJ3	IGLC3			

**Sample**	**clonotype_id**	**igh_cdr3s**	**igh_v–genes**	**igh_j**	**igh_c**	**igl_v–genes**	**igl_j**	**igl_c**	**igk_v_genes**	**igk_j**	**igk_c**
**HC-19**	clonotype916	CARGRRYCSGGSCYEFRRRLWFDPW	IGHV4-34	IGHJ5	IGHM				IGKV1-5	IGKJ3	IGKC
	clonotype1032	CARGDWLTPNLHNYYYGMDVW	IGHV4-34	IGHJ6	IGHM				IGKV1-5	IGKJ1	IGKC
	clonotype1026	CARIRVRPHYYVKSSYFDYW	IGHV4-34	IGHJ4	IGHM				IGKV1-8	IGKJ1	IGKC
	clonotype1400	CARASTYGGNIYYYYGMDVW	IGHV4-34	IGHJ6	IGHM				IGKV1-8	IGKJ2	IGKC
	clonotype428	CARGPSQETLVGFDYW	IGHV4-34	IGHJ4	IGHM				IGKV1-9	IGKJ1	IGKC
	clonotype1185	CARGRPSKTFYDNKYVMDVW	IGHV4-34	IGHJ6	IGHD				IGKV1-9	IGKJ1	IGKC
	clonotype1310	CARGRVVPYVPKYYFDYW	IGHV4-34	IGHJ4	IGHD				IGKV1-16	IGKJ2	IGKC
	clonotype451	CAREKVNPLQSFDYW	IGHV4-34	IGHJ4	IGHM				IGKV1-17	IGKJ1	IGKC
	clonotype1677	CAREVEYCGGDCHWFDPW	IGHV4-34	IGHJ5	IGHM				IGKV1-17	IGKJ4	IGKC
	clonotype1957	CARGLGYGGNSRFDYW	IGHV4-34	IGHJ4	IGHM				IGKV1D-39	IGKJ1	IGKC
	clonotype356	CARGFWYSSPPVDW	IGHV4-34	IGHJ4	IGHM				IGKV1D-39	IGKJ1	IGKC
	clonotype1416	CARGMSPYSGHNNWAFDYW	IGHV4-34	IGHJ4	IGHM				IGKV1D-39	IGKJ2	IGKC
	clonotype38	CARVSGYCSSTSCYRGYYYYGMDVW	IGHV4-34	IGHJ6	IGHM				IGKV1D-39	IGKJ2	IGKC
	clonotype61	CARGLLGISGMDVW	IGHV4-34	IGHJ6	IGHM				IGKV1D-39	IGKJ1	IGKC
	clonotype189	CARRLPRVVGSSVDAFDIW	IGHV4-34	IGHJ3	IGHD				IGKV1D-39	IGKJ1	IGKC
	clonotype553	CARGEYYYDSSGYYYYYYGMDVW	IGHV4-34	IGHJ6	IGHD				IGKV1D-39	IGKJ1	IGKC
	clonotype81	CARGKMAVAGRNSPDFDYW	IGHV4-34	IGHJ4	IGHM				IGKV2-24	IGKJ4	IGKC

	clonotype1255	CARKNLKIGRGYSYGPTRYFDYW	IGHV4-34	IGHJ4	IGHM				IGKV2-28	IGKJ1	IGKC
	clonotype499	CARSLFDIW	IGHV4-34	IGHJ3	IGHM				IGKV2D-29	IGKJ4	IGKC
	clonotype374	CARSLFDIW	IGHV4-34	IGHJ3	IGHM				IGKV2D-29	IGKJ4	IGKC
	clonotype2094	CARGRRIVSGYSSGWYRPRYYFDYW	IGHV4-34	IGHJ4	IGHM				IGKV2D-29	IGKJ2	IGKC
	clonotype1522	CARGRGDYRTSTTDYYYGMDVW	IGHV4-34	IGHJ6	IGHM				IGKV2D-29	IGKJ4	IGKC
	clonotype779	CARGLGTTKRRTGFDIW	IGHV4-34	IGHJ3	IGHM				IGKV3-11	IGKJ4	IGKC
	clonotype911	CASLNIAAHGW	IGHV4-34	IGHJ4	IGHM				IGKV3-11	IGKJ4	IGKC
	clonotype243	CARVAIAVSYYFDYW	IGHV4-34	IGHJ4	IGHM				IGKV3-15	IGKJ4	IGKC
	clonotype105	CARGEYYYDSSGYYYYYYGMDVW	IGHV4-34	IGHJ6	IGHM				IGKV3-15	IGKJ1	IGKC
	clonotype549	CARGFDVDIVATMSTFRAFDIW	IGHV4-34	IGHJ3	IGHD				IGKV3-15	IGKJ5	IGKC
	clonotype608	CARVPGAFDIW	IGHV4-34	IGHJ3	IGHM				IGKV3-20	IGKJ2	IGKC
	clonotype1811	CARGGYYDSSGYPTPGWGFFDYW	IGHV4-34	IGHJ4	IGHM				IGKV3-20	IGKJ2	IGKC
	clonotype102	CARGRRPYYLGVFDPW	IGHV4-34	IGHJ5	IGHM				IGKV3-20	IGKJ1	IGKC
	clonotype391	CARAYCSNISCYPYNWFDPW	IGHV4-34	IGHJ5	IGHM				IGKV3-20	IGKJ1	IGKC
	clonotype330	CASSDVRTVTTRPHYYYYYYGMDVW	IGHV4-34	IGHJ6	IGHM				IGKV3-20	IGKJ1	IGKC
	clonotype1585	CARGPGSSSPYRKYYFDYW	IGHV4-34	IGHJ4	IGHM				IGKV3D-15	IGKJ4	IGKC
	clonotype1057	CARGQHQYYYGSGSPGFDYW	IGHV4-34	IGHJ4	IGHM				IGKV3D-15	IGKJ3	IGKC
	clonotype77	CARGRHGSREIGGWFDPW	IGHV4-34	IGHJ5	IGHM				IGKV3D-15	IGKJ1	IGKC
	clonotype1452	CARGYGPRADQMIVVVITGSSWFDPW	IGHV4-34	IGHJ5	IGHM				IGKV3D-15	IGKJ5	IGKC
	clonotype1535	CARRVGYCSSTSCYHPPSNWYFDLW	IGHV4-34	IGHJ2	IGHM				IGKV4-1	IGKJ3	IGKC
	clonotype576	CARGKRGYSYGFWAFDIW	IGHV4-34	IGHJ3	IGHM				IGKV4-1	IGKJ2	IGKC
	clonotype2001	CAGGYSSGWIANLDYW	IGHV4-34	IGHJ4	IGHM				IGKV4-1	IGKJ4	IGKC
	clonotype293	CTRGEDYYKQGYW	IGHV4-34	IGHJ4	IGHM				IGKV4-1	IGKJ1	IGKC
	clonotype1566	CARALPPVEMATISRDGAGYYFDYW	IGHV4-34	IGHJ4	IGHM				IGKV4-1	IGKJ4	IGKC
	clonotype1359	CARAVPQRIAVAGTRVSPVWFDPW	IGHV4-34	IGHJ5	IGHM				IGKV4-1	IGKJ1	IGKC
	clonotype588	CARAPSIRVVVAATGMWFDPW	IGHV4-34	IGHJ5	IGHD				IGKV4-1	IGKJ1	IGKC
	clonotype175	CARESSSSVVDYW	IGHV4-34	IGHJ4	IGHM	IGLV1-40	IGLJ2	IGLC2			
	clonotype2007	CARARDIVVVVAATHFDYW	IGHV4-34	IGHJ4	IGHM	IGLV1-40	IGLJ2	IGLC2			
	clonotype1332	CARGTAGDSTPKDWFDPW	IGHV4-34	IGHJ5	IGHM	IGLV1-40	IGLJ3	IGLC3			
	clonotype669	CARGQADSRIAARPDWVPRGVWFDPW	IGHV4-34	IGHJ5	IGHM	IGLV1-40	IGLJ3	IGLC3			
	clonotype194	CARGVGGIAAANYRMDVW	IGHV4-34	IGHJ6	IGHM	IGLV1-40	IGLJ2	IGLC2			
	clonotype521	CARIIVVVPAAIYGMDVW	IGHV4-34	IGHJ6	IGHM	IGLV1-40	IGLJ3	IGLC3			
	clonotype2098	CARGPVHPEKRGGEDYFDYW	IGHV4-34	IGHJ4	IGHM	IGLV1-44	IGLJ2	IGLC2			
	clonotype1759	CARAIGGYYRFVYYFDYW	IGHV4-34	IGHJ4	IGHM	IGLV1-47	IGLJ3	IGLC3			
	clonotype92	CARDPEYYYDSSGYFGPEYYFDYW	IGHV4-34	IGHJ4	IGHM	IGLV1-47	IGLJ3	IGLC3			
	clonotype1448	CARGGRYYTKEELRRGSRFYFDYW	IGHV4-34	IGHJ4	IGHM	IGLV1-51	IGLJ2	IGLC2			
	clonotype930	CARALQGSSGYYYRCPYFDYW	IGHV4-34	IGHJ4	IGHM	IGLV1-51	IGLJ3	IGLC3			
	clonotype625	CAREGDPGIAVAGFDYW	IGHV4-34	IGHJ4	IGHM	IGLV2-11	IGLJ3	IGLC3			
	clonotype965	CARSIAAAGTSGVRYFDLW	IGHV4-34	IGHJ2	IGHM	IGLV2-14	IGLJ2	IGLC2			
	clonotype1396	CARGKLGPYSYAPPHSFDFDYW	IGHV4-34	IGHJ4	IGHM	IGLV2-14	IGLJ2	IGLC2			
	clonotype734	CARELPYGVVIPHYFDYW	IGHV4-34	IGHJ4	IGHM	IGLV2-14	IGLJ3	IGLC3			
	clonotype895	CARGIAWGYDSSGYHKGYFDYW	IGHV4-34	IGHJ4	IGHM	IGLV2-14	IGLJ3	IGLC3			
	clonotype756	CAGALPELPPYAFDYW	IGHV4-34	IGHJ4	IGHD	IGLV2-18	IGLJ3	IGLC3			
	clonotype1972	CARSWENSSGYYYDYW	IGHV4-34	IGHJ4	IGHM	IGLV2-23	IGLJ2	IGLC2			
	clonotype557	CARVHQLVDIRDWFDPW	IGHV4-34	IGHJ5	IGHM	IGLV3-1	IGLJ2	IGLC2			
	clonotype1717	CARGYIVVVPAATVNYFDYW	IGHV4-34	IGHJ4	IGHM	IGLV3-19	IGLJ2	IGLC2			
	clonotype1466	CARGRVIGLGSRYYDFWSGSHMDVW	IGHV4-34	IGHJ6	IGHM	IGLV3-19	IGLJ2	IGLC2			
	clonotype1549	CARGHFHPLDTSGGSSRSWWFDPW	IGHV4-34	IGHJ5	IGHD	IGLV3-19	IGLJ2	IGLC2			
	clonotype1165	CARGTPPLSSGWYRGWFDPW	IGHV4-34	IGHJ5	IGHM	IGLV3-9	IGLJ2	IGLC2			
	clonotype1550	CARGALNLFVYLNRQTPGKRGIFDYW	IGHV4-34	IGHJ4	IGHM	IGLV9-49	IGLJ3	IGLC3			

Sequences from each sample are organized by VK followed by VL gene expression
